# Macroalgal cover on coral reefs: Spatial and environmental predictors, and decadal trends in the Great Barrier Reef

**DOI:** 10.1371/journal.pone.0279699

**Published:** 2023-01-20

**Authors:** Katharina E. Fabricius, Kerryn Crossman, Michelle Jonker, Mathieu Mongin, Angus Thompson

**Affiliations:** 1 Australian Institute of Marine Science, Townsville, Queensland, Australia; 2 CSIRO Oceans and Atmospheric Processes, Hobart, Tasmania, Australia; University of Technology Sydney, AUSTRALIA

## Abstract

Macroalgae are an important component of coral reef ecosystems. We identified spatial patterns, environmental drivers and long-term trends of total cover of upright fleshy and calcareous coral reef inhabiting macroalgae in the Great Barrier Reef. The spatial study comprised of one-off surveys of 1257 sites (latitude 11–24°S, coastal to offshore, 0–18 m depth), while the temporal trends analysis was based on 26 years of long-term monitoring data from 93 reefs. Environmental predictors were obtained from *in situ* data and from the coupled hydrodynamic-biochemical model eReefs. Macroalgae dominated the benthos (≥50% cover) on at least one site of 40.4% of surveyed inshore reefs. Spatially, macroalgal cover increased steeply towards the coast, with latitude away from the equator, and towards shallow (≤3 m) depth. Environmental conditions associated with macroalgal dominance were: high tidal range, wave exposure and irradiance, and low aragonite saturation state, Secchi depth, total alkalinity and temperature. Evidence of space competition between macroalgal cover and hard coral cover was restricted to shallow inshore sites. Temporally, macroalgal cover on inshore and mid-shelf reefs showed some fluctuations, but unlike hard corals they showed no systematic trends. Our extensive empirical data may serve to parameterize ecosystem models, and to refine reef condition indices based on macroalgal data for Pacific coral reefs.

## Introduction

**Fig 1:**
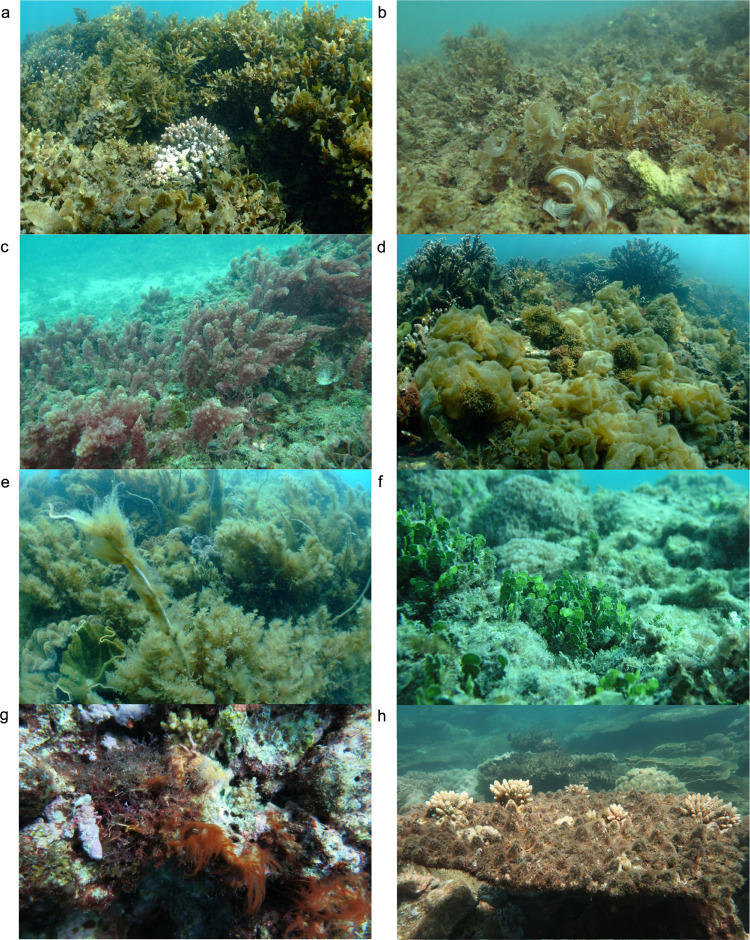
Examples of the major reef algal assemblages on the Great Barrier Reef. (**a, b)** In turbid waters especially on shallow wave-exposed inshore sites, *Sargassum*, *Padina*, *Lobophora* and other members of the class Phaeophyceae can dominate reef communities (**a:** Magnetic Island 2020; **b:** Havannah Reef 2007). (**c)** The red alga *Asparagopsis* can be common on disturbed high-current reef sites (Havannah Reef). (**d)** Relatively short-lived blooms of ephemeral Phaeophyceae can create anoxic conditions that may kill corals (Magnetic Island 2019). (**e)** Spring blooms of filamentous algae predominantly occur in high-nutrient environments and can be lethal for other reef benthos especially during senescence (Dingo Beach, Whitsundays). (**f)** On offshore reefs, the calcareous macroalga *Halimeda* can occupy extensive areas (Green Island). (**g)** Cyanobacteria may cover large reef areas after disturbance, but these blooms are functionally different from macroalgae and are excluded here from ‘total macroalgal cover’ (John Brewer Reef). (**h)** Turf algae, rather than macroalgae, occupy GBR reefs after disturbances in many environmental conditions (see Results) (Russell Island). Photos: **a,c,d,e,g,h:** K. Fabricius, AIMS; **b,f:** LTMP, AIMS.

Macroalgae are an integral component of benthic ecosystems. They are important primary producers and the foundation for complex food chains, serve as habitat for many invertebrates and juvenile fishes, and some are of economic value [1, 2]. However, on coral reefs, high macroalgal abundances are typically considered detrimental, as certain macroalgae can negatively affect coral health, larval settlement and juvenile survival, and hence hinder reef recovery after a disturbance (Fig 1; [3–5]). Dense canopy-forming macroalgae such as the abundant *Sargassum* spp. with perennial holdfasts may affect coral populations by shading, space occupancy, abrasion, sediment trapping, and the release of dissolved organic carbon and secondary metabolites (Fig 1A–1C; [6, 7]). Dense mats of ephemeral algae may also restrict gas exchange and lead to hypoxic conditions with high CO_2_ and reduced pH around corals, especially when such mats senesce and collapse (Fig 1E and 1F). For these reasons, measures of benthic macroalgal cover are often used as one component of indicators for coral reef ecosystem condition, with a more negative score as macroalgal cover increases, but typically without consideration of expected values at a given location [4, 8–10], but see [11].

Coral reef inhabiting macroalgae comprise many hundreds of functionally diverse species in about 40 orders from three major phyla: Rhodophyta (red algae), Ochrophyta (predominantly Phaeophyceae, brown algae), and Chlorophyta (green algae) [12, 13]. In the present study, the term ‘total macroalgal cover’ (henceforth MA) was defined to include all corticated, branched, leathery articulated or jointed upright benthic macroalgae, as well as fleshy prostrate or foliose algae such as *Lobophora*, *Padina*, *Ulva* or *Dictyota* [12–14]. Excluded are the morphologically simpler filamentous algal turfs (typically <10 mm in height), large cyanobacteria colonies, colonial chrysophytes, and calcareous and non-calcareous crustose coralline algae, due to their functional differences (Fig 1), [12–14]., Due to the large-scale nature of this study, small cryptic macroalgae that are almost ubiquitous in gaps within the complex reef matrix were not considered, and canopy heights were not measured.

Some reef habitats show apparently persistent high MA [15, 16], but it is often unclear whether macroalgal dominance on coral reefs represents a ‘natural’ reef state, or whether it is attributable to intensifying disturbances from climate change, eutrophication and sedimentation, overfishing, and other human activities [17]. Shifts from a coral to a macroalgal dominated reef state can lead to novel reef ecosystem types with compromised capacity to revert to coral dominance. Phase shifts from corals to macroalgae are especially well described for some Caribbean coral reefs where herbivore abundances are low from overfishing and urchin diseases [18, 19]. Phase shifts appear more infrequent and short-lived in the Indo-Pacific including the Australian Great Barrier Reef (GBR) [20]. Although the comparison between past studies suffers from inconsistency in definitions and short study periods, only few published examples exist of phase shifts from corals to macroalgae that persisted locally beyond 5 years in that region [20].

The persistence of phase shifts on coral reefs is often attributed to top-down controls by herbivores [21] or bottom-up controls by nutrients [4, 22, 23]. Herbivory by fishes and invertebrates (e.g., sea urchins, large crabs, gastropods or small amphipods can prevent the establishment of macroalgal dominance, especially by suppressing macroalgal recruitment, supporting the protection of herbivores and of trophic structures in coral reefs [14, 24–26]). MA abundances also increase in areas of poor water quality, which emphasizes the need for effective water quality guidelines [27, 28].

Where coral-macroalgal phase shifts persist despite conventional reactive management actions of herbivore protection and water quality management, proactive MA removal or other forms of interventions, are increasingly considered with the aim to accelerate reef recovery, counteract declining coral cover or support coral restoration activities [25, 29, 30]. However, as proactive interventions are generally costly, they need to be underpinned by sound ecological data and ecosystem models, to inform about what type of intervention would appear beneficial in which location and under what environmental conditions [31]. Empirical data are also needed to improve the use of MA as a metric in reef health indices, taking into consideration natural spatial gradients and environmental drivers for these communities to detect deviations from ‘desirable’ ecosystem state.

**Fig 2:**
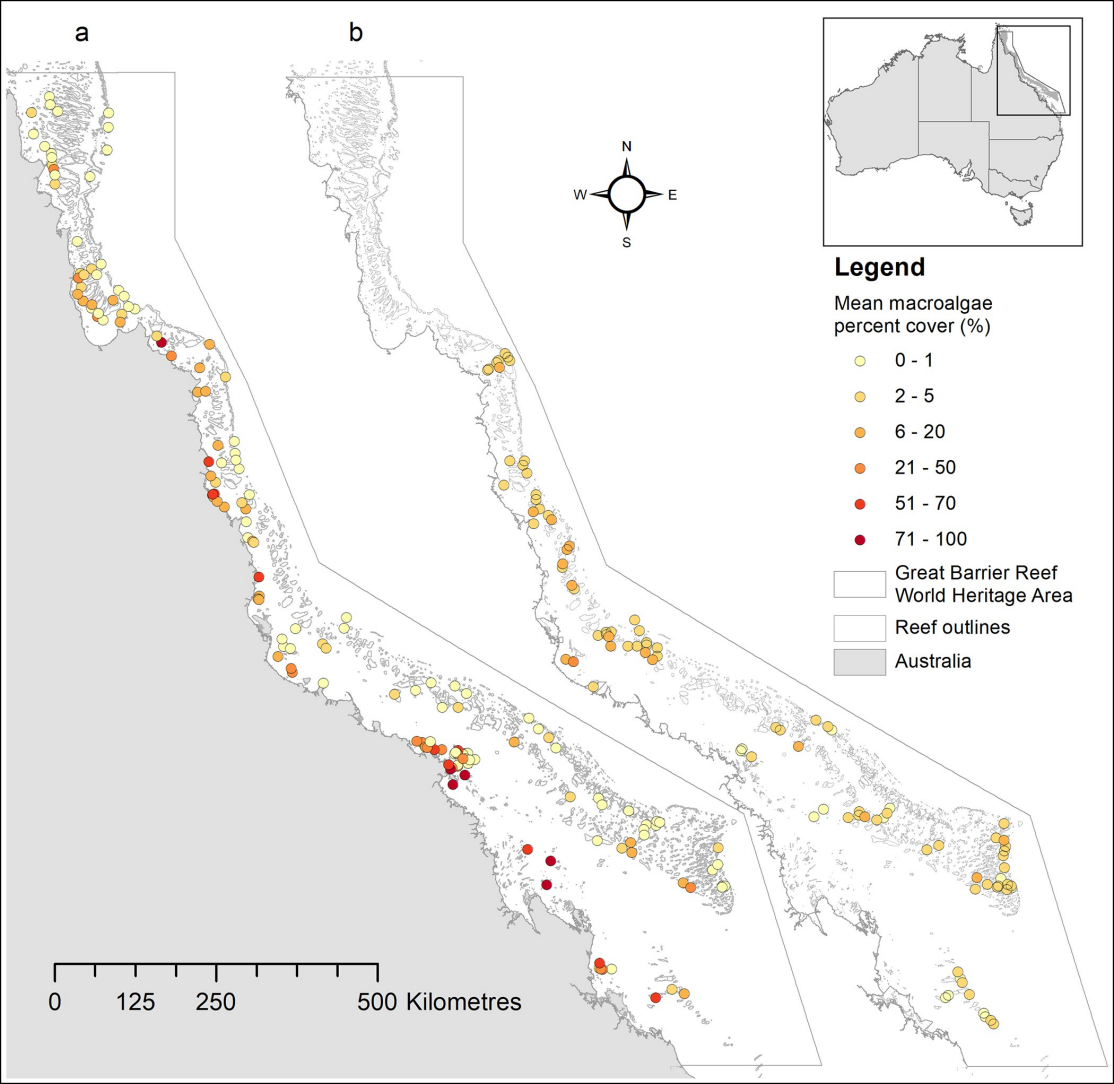
Macroalgal survey locations on the Great Barrier Reef, north-eastern Australia. Colour shading indicates mean macroalgal cover. **a.** One-off surveys, here shown with values for 2 m depth, **b.** AIMS Long-Term Monitoring Program at 6–8 m depth. Coastline: Reprinted under a Creative Commons Attribution 4.0 International license, with permission from Australian Bureau of Statistics, original copyright 2011. Reef outlines: Reprinted under a CC BY license with permission from the Great Barrier Reef Marine Park Authority, original copyright 1998. Great Barrier Reef World Heritage Area: Reprinted from Australian Government Department of Climate Change, Energy, the Environment and Water under a CC BY license, with permission from Australian Government Department of Climate Change, Energy, the Environment and Water, original copyright 2022.

The GBR is a vast coral reef system that extends from 10.5–24° S latitude and covers 348,000 km^2^. It is composed of almost 3000 coral reefs that encompass a wide range of reef types, from tropical to subtropical and from clearwater offshore to turbid inshore reefs with and without exposure to terrestrial runoff of nutrients and sediments. Previous studies on GBR macroalgae have mostly been conducted at individual reefs or small reef clusters, with few exceptions [11, 32, 33]. The objectives of our study were to investigate the likely spatial and environmental predictors and long-term temporal trends of MA on the GBR. The spatial component of our study was based on a large one-off survey data set of 1257 sites covering most habitat types across and along the GBR from 0 to 18 m depth (Fig 2). Site-specific nutrient, sediment, light and current estimates were available for the GBR through models. Herbivory was not included as predictors since no consistent empirical or modeled data on herbivore abundances and their feeding rates exist for all study sites. The data on temporal dynamics were based on 26 years of annual or biennial long-term monitoring data from up to 93 reefs. Our data in combination inform about the main spatial and environmental predictors of MA in coral reefs, where to expect macroalgal dominance and its association with hard coral cover, and on long-term temporal dynamics in MA across the GBR.

## Methods

### Field methods

One-off surveys were conducted on 1257 transects at 324 sites on 163 reefs between latitude 11.0° to 23.6°S (Fig 2A). Reef types included coastal fringing, island fringing and platform at varying distance reefs across the continental shelf. One to three sites were surveyed across reef habitats (windward front, leeward back, flank, lagoon reef sites), each with up to 5 depth zones (deep, mid and upper reef slopes at 18–13 m, 13–8 m and 8–3 m, reef crest at 3–1 m, and the outer edge of reef flats). The surveys were based on rapid ecological swim surveys [34], in which a single observer (KEF) dived along a 200 to 300 m long and 2–3 m wide transect, recording visual estimates of percent cover of total macroalgae (MA), together with the percent cover of other main benthos groups (turf algae, crustose coralline algae, cyanobacteria, hard corals, octocorals, other benthic invertebrates, sand and rubble. Rapid ecological swim surveys are used to cover larger and hence more representative reef areas than photo or belt transects, yet with greater taxonomic detail than can be obtained by manta tow methods [35, 36]. The surveys were conducted between 1997 and 2008, a time when GBR-wide hard coral cover averaged 20–25% [37]. Although not recent, the data are ecologically valuable due to their large spatial coverage along complex environmental gradients.

Long-term trends in GBR macroalgal cover were assessed by analyzing 26 years (1995–2021) of underwater image-based survey data from the Long-Term Monitoring Program of the Australian Institute of Marine Science (LTMP) [38]. Data are from 6–8 m mean depth on the northeastern flanks of 93 reefs between latitude 14.6°S to 23.9°S (Fig 2B). Reefs were grouped into three geomorphological groups (inshore reefs exposed to resuspension of coastal sediments, mid-shelf, and offshore reefs along the seaward margin of the continental shelf). Between 1995 and 2005, 47 reefs were surveyed annually, including eleven inshore reefs. From 2006, 93 reefs were surveyed biennially (year 1: the 47 previous reefs, year 2: 46 additional reefs, which did not include additional inshore reefs). At each reef, benthos was classified along five permanently marked 50-m transects at each of three sites. Benthos was identified to the finest taxonomic resolution possible under five fixed points on each of forty images per transect (n = 200 points per transect [38]). Switching from video to high resolution photographic records in 2006 led to small adjustments in the definitions of the category ‘macroalgae’, especially the exclusion of thick cyanobacterial mats.

The work was conducted under the following research permits by the Great Barrier Reef Marine Park Authority: the one-off surveys were covered by the general Research Permit granted to the Australian Institute of Marine Science (Permit Number G06/15571.1). The Long-Term Monitoring Program field activities were covered by Permit Numbers G18/40177.1, G12/34872.1, G06/19994.1, G03/10944.1 and G00/462.

### Environmental predictors

To obtain long-term mean values reflecting persistent environmental conditions, site- and depth specific environmental variables were derived from ‘eReefs’. We used version GBR4 H2.0 BGC3.3 of the three-dimensional coupled hydrodynamic, biogeochemical, optical and sediment model for the GBR reef matrix (4 km horizontal scale, 41 depth levels, hourly intervals from December 2010 –April 2021) [39, 40]. The eReefs model is forced using wind, rainfall, pressure, air and dew-point temperature and cloud amount from the Bureau of Meteorology’s ACCESS-R (http://www.bom.gov.au/nwp/doc/access/NWPData.shtml). Nutrient and sediment discharges from 22 rivers are calculated from a customized version of the SOURCE catchment model [41]. The biogeochemical model simulates optical, nutrient, detritus, chemical and sediment dynamics. The model variables are described in [40], the carbon chemistry in [42], and the eReefs simulation skills and comparison with *in situ* observations in [39]. eReefs estimates were obtained for the closest 4 km grid point and the nearest depth for each one-off survey site. All values were averaged over months, and then across the whole simulation period (12/2010–04/2021). Simulated concentrations of dissolved inorganic carbon (DIC) and total alkalinity (A_T_) are driven by photosynthesis/respiration and calcification/dissolution processes on the reefs as well as by carbon equilibrium with the atmosphere and riverine inputs. These values, together with salinity and temperature, were used to calculate aragonite and calcite saturation state (Ω_ar_, Ω_ca_, dimensionless), pH and pCO_2_ as proxy for all other seawater carbon chemistry variables, using the R library SeaCarb [43]. S1 Table in S1 File lists all variables included in the exploratory and final models.

Additionally, some finer-scale environmental data were visually estimated for each site during the one-off surveys: the amount of sediment deposited on the reef substratum rated on a 4-point scale, typical wave exposure rated on a 5-point scale, reef slope angle, and reef rugosity rated on a 4-point scale (S1 Table in S1 File; [34]).

### Statistical methods

Physical and ecological gradients typically run steeply across and, to a lesser degree, along the long and narrow GBR, which tilts ~45° to the geodesic system. To improve spatial analyses, the latitude/longitude data were converted into relative distance across and along the GBR Marine Park (henceforth: ‘*across*’, ‘*along*’; [27]). Across is defined as the proportional distance of a site from the coast (*across* = 0) to the outer edge of the continental shelf (*across* = 1). Ditto for *along* (southern end: *along* = 0, north: *along* = 1). All analyses used the statistical software R Version 3.4.3 [44]. The one-off survey and LTMP data were not combined in the analyses, due to differences in depths, reef zones and survey methods.

One-off surveys: Spatial and environmental predictors for macroalgal cover. To find the strongest predictors of MA in the one-off data, variation in MA was related to the spatial and environmental variables (S1 Table in S1 File) using non-parametric aggregated boosted regression tree analyses (ABT) (R package ‘abt’ [45]). This machine learning method is based on ensembles of classification and regression trees, with algorithms learning the relationship between a predictor and its response. Predictor variables can be numeric or categorical and measured at different scales, and model outcomes are insensitive to predictor transformations and outliers, hence boosted regression tree analyses are advantageous for models with complex, non-linear interactions [45]. A spatial and geomorphological ABT (henceforth ‘spatial model’), and a physical and biochemical environmental ABT model (henceforth ‘environmental model’) were run separately, due to the spatially confounded nature of some of the environmental variables. For example, there is a ~3°C mean temperature difference between the northern and southern GBR, mean tides range from <1.5 m to a maximum of 6.7 m (~22°S), water clarity and other variables steeply change from the coast to the outer continental shelf, and PAR is confounded with depth at the within-reef scale. The models were first run with all spatial or environmental predictors included, including their interactions. To select the best models, the prediction error was minimized based on cross-validation, also investigating data distributions and interactions. The weakest predictors were sequentially removed (S1 Table in S1 File), monotonicity of the relationships of the remaining variables were investigated, and interaction levels reduced where prediction errors did not substantially increase. Results were presented as partial dependency plots, displaying the effect of each covariate as the effects of all other covariates held constant. The final spatial model included three-way interactions, the final environmental model was an additive main effects model.

Interactions between macroalgae and other reef benthos. To explore the relationship between MA and hard coral cover, Bayesian hierarchical linear models were fit using the R package INLA (Integrated Nested Laplace Approximation [46]). Exploratory data analyses revealed that there were a high percentage of 0 macroalgae counts (resulting in zero-inflation) and that the zero counts did not seem to be randomly distributed throughout the design. Specifically, zero counts were particularly notable on offshore reefs and in deeper habitats. The observed macroalgae counts were considered to have arisen via two processes. Firstly, a Bernoulli process governing whether macroalgae were present (or at least present in sufficient enough quantity so as to be detectable) and, secondly, a count process that governed the abundance of macroalgae given the conditions were conducive of macroalgae presence. As such, macroalgae relationships were explored via hurdle-like models in which the presence/absence of macroalgae was first explored using a binomial (Bernoulli) distribution (logit link function) (S1 Fig in S1 File), and then the positive abundance of macroalgae was explored using a beta distribution (logit link function) and an observation-level random effect to account for overdispersion. In each case, the models included the population effects of percentage hard coral cover, depth and shelf position as well as the hierarchical structure of Transects nested within Sites nested within Reefs. Fixed effects and hyperparameters for each model are provided in the supplementary materials (S2-S5 Tables in S1 File). All models were validated via DHARMa residual diagnostics [47] (S2 & S3 Figs in S1 File). Modelled higher posterior density (HPD) means and 95% Bayesian Credible Intervals (CIs) for macroalgal cover along a hard coral cover gradient are presented for each combination of shelf position and depth.

The relationships between MA and other aspects of the reef communities and all spatial and environmental variables were integrated and visualized with a redundancy analysis (RDA, R package ‘vegan’ [48]), with both species and environmental vectors scaled by their eigenvalues. To assess the significance for each predictor in the presence of all other predictors, a permutation test (non-sequential partial model, 999 iterations) was applied to the scaled RDA results, permuting the residuals of the community data conditional to the other variables being held constant [49, 50].

Temporal trends in macroalgal cover. Rates of temporal changes in MA in the Long-term Monitoring Program (LTMP) data were estimated using a Bayesian hierarchical linear INLA model [46]. Data were fit via a binomial distribution with a logit link, and the two components of the response variable were a count of macroalgae points and a total number of classified benthic points per transect. First, temporal changes in MA were estimated for the whole GBR, and then for inshore, mid-shelf and offshore LTMP reefs. ‘Year’ was specified as a population effect and ‘month’ was included as a random effect to account for seasonal variation in macroalgae growth. ‘Shelf’, ‘Reef’, ‘Site’ and ‘Transect’ were included as nested, random effects, along with an observation level random effect to account for overdispersion. The model was repeated with an interaction for the population effects of ‘Year’ and ‘Shelf’, and the random effect of ‘Shelf’ removed. Data for the sample year 1999 were excluded from both the GBR and shelf-level models due to a quality control issue regarding the identification of algae at some inshore and mid-shelf reefs that is yet to be resolved. Last, temporal trends in MA were modelled for individual reefs. Models were again fit with a binomial distribution (logit link function) with the population effects of ‘Year’, a random effect of ‘month’, an observation-level random effect, and the hierarchical structure of Transects nested within Sites nested within Reefs. Fixed effects and hyperparameters for each model are provided in the supplementary materials (Whole GBR model—S6 & S7 Tables in S1 File; cross-shelf model–S8 & S9 Tables in S1 File; models for individual reefs–S1 & S2 Tables). All models were validated via DHARMa residual diagnostics (Whole GBR model–S4 Fig in S1 File; cross-shelf model–S5 Fig in S1 File; individual reef models–S6A-S6L Fig in S1 File). Modelled HPD means and 95% CIs for MA are presented for the whole GBR, three shelf positions, and individual reefs. For clarity, trends are only shown for the 12 reefs with the highest modelled MA values in anyone year.

## Results

### One-off surveys: Spatial and environmental predictors for total macroalgal cover

Across the 1257 one-off survey sites spanning 11 years (Fig 2), total cover of macroalgae (MA) averaged 9.3% ± 0.56 SE but was highly variable. MA exceeded 50% and 70% cover in 7.2% (91/1257) and 4.5% of all sites, respectively. Sites with ≥50% MA were mostly found on inshore reef flat and crests; only 16% of the sites with ≥50% MA were inshore reef slopes, 5% were mid-shelf, and none were outer-shelf sites. Overall, 40.4% and 29.8% of the surveyed inshore reefs had at least one site with ≥50% and ≥70% MA, respectively; this percentage was 4.2% and 1.0% for mid-shelf reefs. Hard coral and octocoral cover averaged 23.5% ± 0.57 and 10.3% ± 0.39, respectively. The remaining space was covered by turf (34.3% ± 0.62) and coralline algae (8.9% ± 0.39) and other reef invertebrates or sand (~12%).

**Fig 3:**
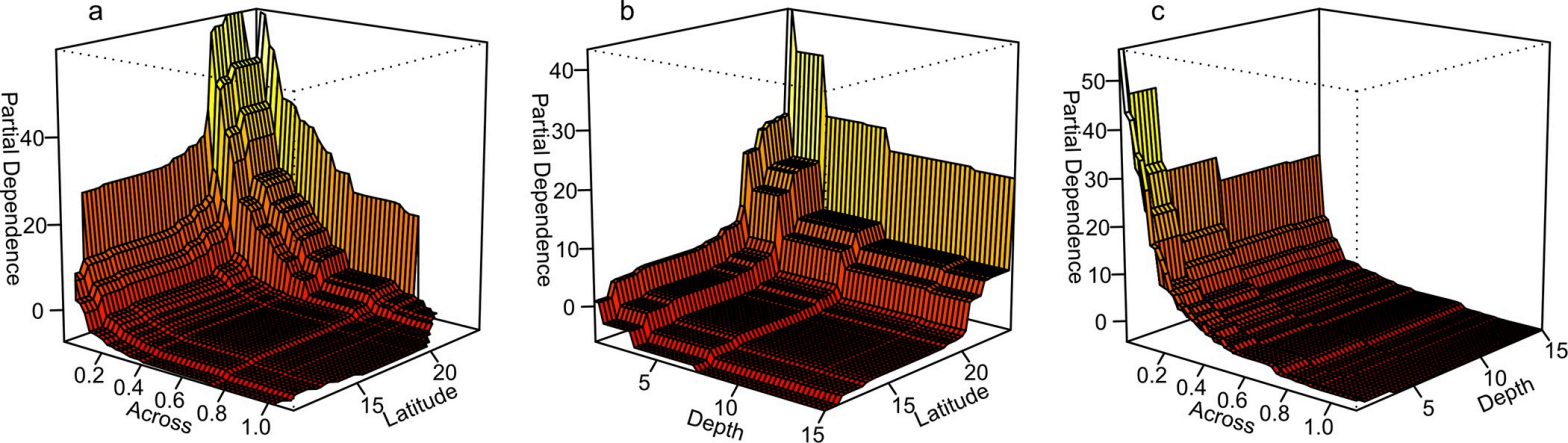
Total macroalgal cover (MA) as a function of the three strongest spatial variables. Partial dependence plots, showing changes in MA as deviation from their mean value of 9.3% (vertical axis and colour contours, in percent cover) attributable to the three interacting spatial predictors: relative distance *across* the continental shelf (0 = coast, 1 = offshore), latitude (°S), and depth (m) (**a–c**). Relative percentual influence of the predictors to main effects without interaction: *across* = 40.8%, latitude = 26.4%, depth = 20.0%, within-reef location = 6.71%, reef type = 6.10%.

The spatial ABT model showed the most important predictors for MA to be cross-shelf position, latitude and depth (Fig 3). MA increased across the continental shelf from offshore towards the coast at all latitudes, with the steepest increases nearest the coast (<0.15 *across*; Figs 2 and 3) and south of latitude 20°S. When all other factors were held constant, the difference in mean MA between offshore and coastal reefs was ~35% MA cover. Additionally, MA increased with latitude by ~25% cover, with steepest increases south of latitude 20°S on inshore and mid-shelf reefs. MA additionally increased monotonically by ~8% cover from deep to shallow water, with greatest changes occurring above 3 m depth near southern coasts. Our preliminary data analyses found habitat type, reef type and season not to be a significant predictor for MA hence these factors were dropped from the final model. Across habitat types, MA was slightly higher on windward compared with leeward sites, and highly variable in reef lagoons and channels. The model remained very similar when *across* was replaced by the 3-level factor ‘shelf position’, or latitude was exchanged against *along*, confirming the robustness of the spatial model.

**Fig 4:**
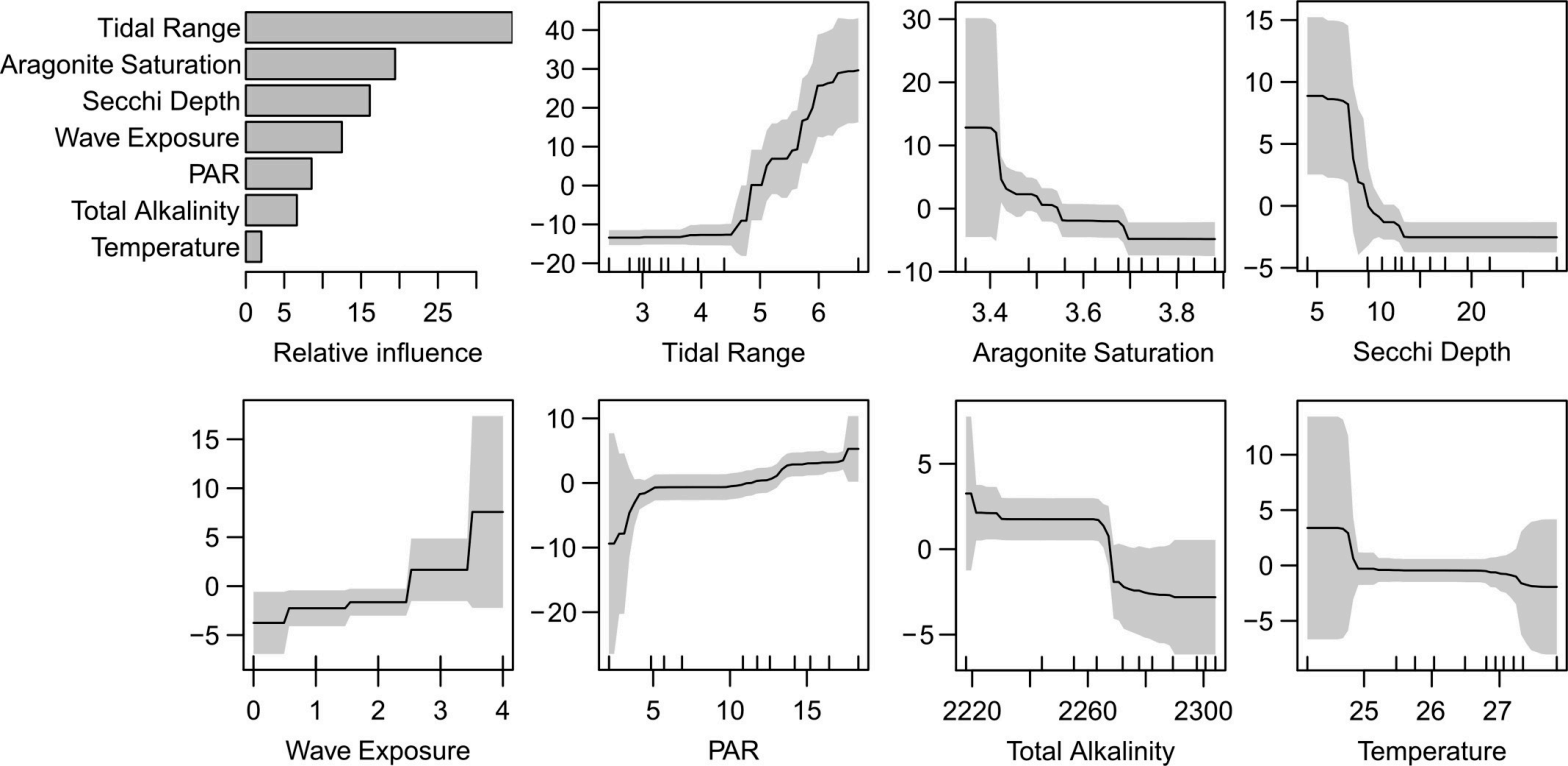
Total macroalgal cover (MA) in response to long-term mean environmental conditions. Partial dependence plots, showing the relative influence (percentage contribution) of each predictor, and the changes in MA to the predictors (solid lines), and their 95% confidence intervals (grey envelopes). The y-axis represents the change in MA relative to the global mean (9.3% cover) attributable to each predictor. The ticks on the x-axis show the deciles of the predictors’ data distributions. Definitions and units of the predictors in S1 Table in S1 File.

The environmental ABT model showed that MA was well predicted by four physical and three biochemical predictors, without interactions (Fig 4). For the factors that varied mostly between (rather than within) reefs, mean tidal range, aragonite saturation state and Secchi depth were the most important predictors, associated with monotonic changes by 42%, ~18% and 12% in mean MA, respectively. For the factors that also varied within reefs, MA additionally increased by >12% each with increasing wave exposure and increasing PAR at the depth of the site (i.e., shallow sites in turbid water). As latitude, temperature, aragonite saturation state and mean tidal range are partially correlated, their relative contribution could not be entirely resolved. When latitude was added to the environmental model, tidal range, aragonite, Secchi depth, waves and PAR remained the most important factors in this expanded model, temperature lost most of its importance and instead latitude was found to be important (relative influence 5.7%), suggesting temperature may be the main reason for the observed latitudinal decline. Similarly, mean total alkalinity and salinity were strongly correlated with cross-shelf position, but all other predictor variables remained important when across was added to the environmental model. Of the correlated carbonate chemistry variables (pCO_2_, DIC, pH, aragonite saturation state), the latter was stronger than the others and hence the only variable retained in the final model. Similarly, of the various measures of water clarity (Secchi depth, Kd, turbidity, PAR at 3.2 m depth), Secchi depth was consistently the strongest predictor. Other predictors (e.g., reef slope angle, mean salinity, mud, total chlorophyll, dissolved inorganic nitrogen, ratio of summer to winter PAR) were of lower importance. Changes in MA were particularly steep in response to three predictors: MA steeply increased towards the coast but remained largely invariant on mid- to outer shelf. MA also steeply increased at >4.5 m tidal range, and at <10 m Secchi depth. The other spatial and environmental gradients were associated with more gradual changes in MA after the other spatial or environmental variables were controlled for.

### Interactions between macroalgae and other reef benthos

**Fig 5:**
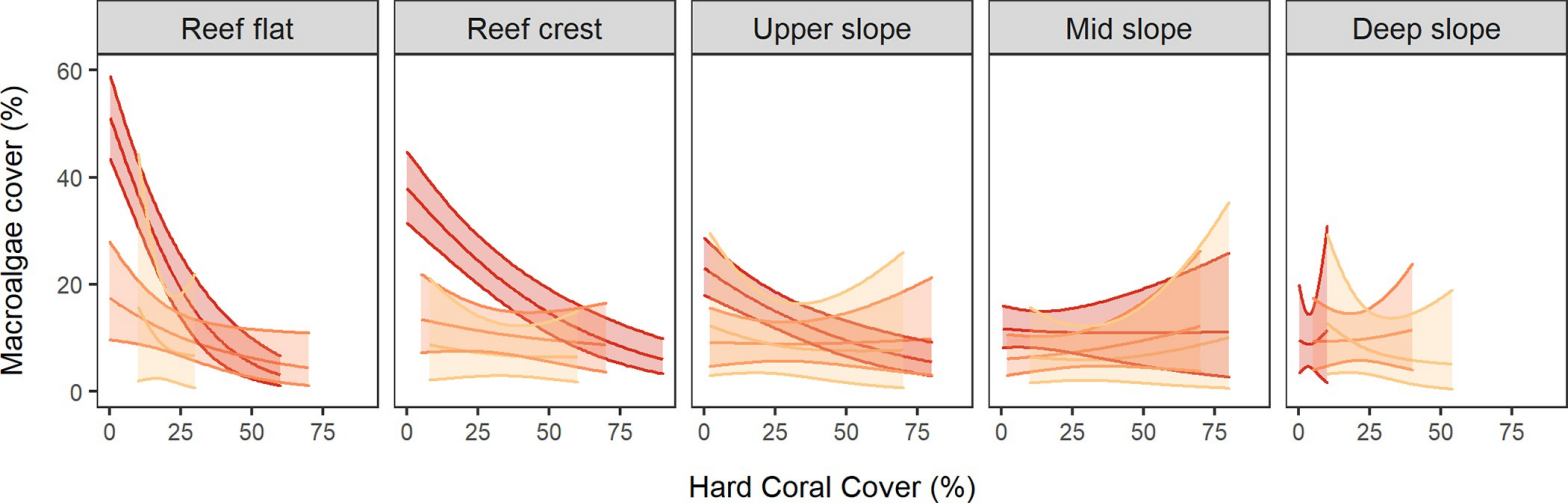
Relationship between macroalgal cover (where MA was present), and hard coral cover. Plots based on the one-off surveys across five depth zones and three shelf positions (Red = Inshore, Orange = Mid-shelf, Light orange = Outer shelf). Solid lines are modelled higher posterior distribution means, shadings are 95% Bayesian Credible Intervals. Statistical differences can be inferred where 95% CIs do not overlap.

The INLA model showed that where macroalgae were present, the relationship between hard corals and macroalgae was most distinct on inshore reefs, particularly in less than 3 m water depth (Fig 5). On inshore reef flats (1 m depth), MA declined from 51% cover where hard coral cover approximated 0.1%, to 3% where hard coral cover reached 60%. This equated to an 8% decline in MA on average for every 10% increase in hard coral cover (note this relationship is not easily resolved, since these two groups are statistically not independent). On inshore reef crests (1–3 m depth), MA was 38% where hard coral cover approximated 0%, and declined at an average rate of 3.5% per 10% increase in hard coral cover. On inshore upper slopes (3–8 m depth), MA was 23% when hard corals were absent and decreased 2% per 10% increase in hard coral cover. On the mid-shelf, MA (where present) was highest on the reef flat (17%), where it declined at an average rate of 2% for every 10% increase in hard coral cover. On the outer-shelf, MA (where present) was highest on the reef flat (15.8%), decreasing at a rate of 4.6% for every 10% increase in hard coral cover over the relatively small range of hard coral cover observed (10–30%). Everywhere else (inshore mid-slope and deep slope habitats, crests and slopes of mid-shelf and outer-shelf reefs), MA was low (<14% where present), with higher uncertainty, and no systematic relationship with changes in coral cover.

**Fig 6:**
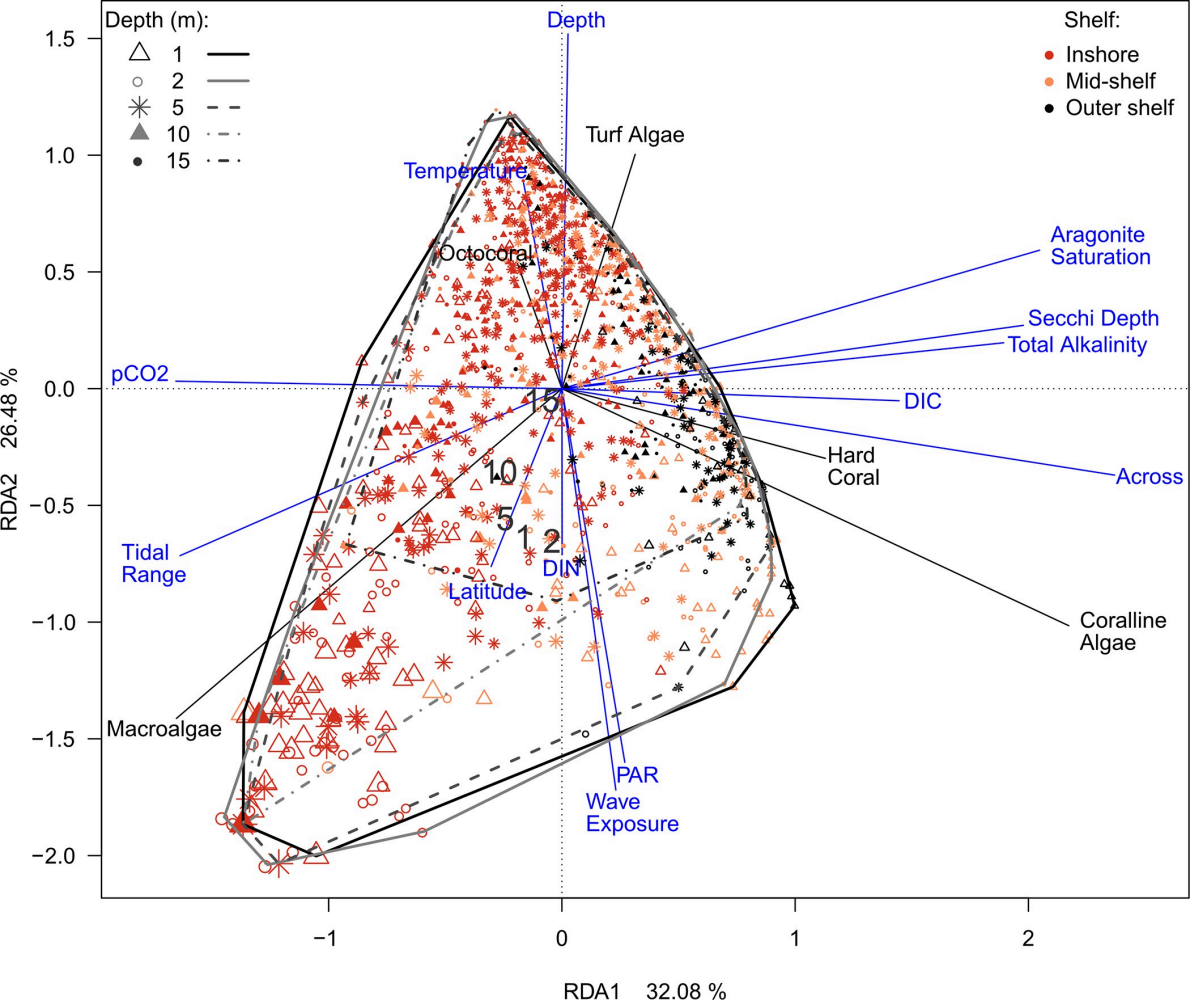
Redundancy analysis, showing the relationships between benthos and predictors. Plot based on the one-off surveys, and the main benthic groups (black arrows: cover of total macroalgae, hard corals, soft corals, coralline algae and turf algae, and their spatial and environmental predictors (blue arrows; definitions in S1 Table in S1 File). Species and environmental data scaled by eigenvalues (significance of relationships: Table 1). Each symbol represents a site, colors indicate shelf position, size increases with MA (range: 0% - 100% cover), and the symbols and hulls denote survey depths.

The redundancy analysis further differentiated the multiple complex relationships between the main benthic groups and the most important spatial and environmental variables (Fig 6). The first two axes represented 58.56% of variance. It confirmed the strong association of MA with high mean tidal range, high wave exposure, a short distance *across* the shelf, shallow depths, high latitude, high mean PAR at the depth of the site and low values of Secchi depth (i.e., shallow and turbid water), low aragonite saturation state (high pCO_2_), and low total alkalinity. In contrast, hard corals and crustose coralline algae were predominantly associated with greater distance *across* the shelf, and high aragonite saturation state / low pCO_2_ while octocorals and turf algae were associated with greater depth and low wave exposure. There was again a negative association between MA and hard coral and octocoral cover, suggesting these three benthic groups occupy somehow contrasting environmental niches. The permutation analysis confirmed the significant relationship of the benthic groups to these factors, with *across* accounting for the greatest F-ratio, followed by three carbonate chemistry variables, depth, temperature and wave exposure (Table 1).

**Table 1:** Significance of the predictors for the community data.

	Df	Variance	F	P
Across	1	0.274	20.58	<0.001
Latitude	1	0.123	9.24	<0.001
Depth	1	0.167	12.54	<0.001
Wave Exposure	1	0.233	17.50	<0.001
Tidal Range	1	0.097	7.31	<0.001
Secchi Depth	1	0.029	2.18	0.073
PAR	1	0.130	9.80	<0.001
Total Alkalinity	1	0.243	18.28	<0.001
DIC	1	0.239	18.00	<0.001
DIN	1	0.068	5.09	<0.001
Temperature	1	0.213	16.02	<0.001
Aragonite Saturation	1	0.229	17.24	<0.001
pCO2	1	0.178	13.39	<0.001
Residual	1243	16.519		

Non-sequential permutation test of RDA results (Fig 6), testing the marginal effects of each predictor variable in the presence of all other predictor variables.

### Long-term monitoring surveys: Temporal trends in macroalgal cover

**Fig 7:**
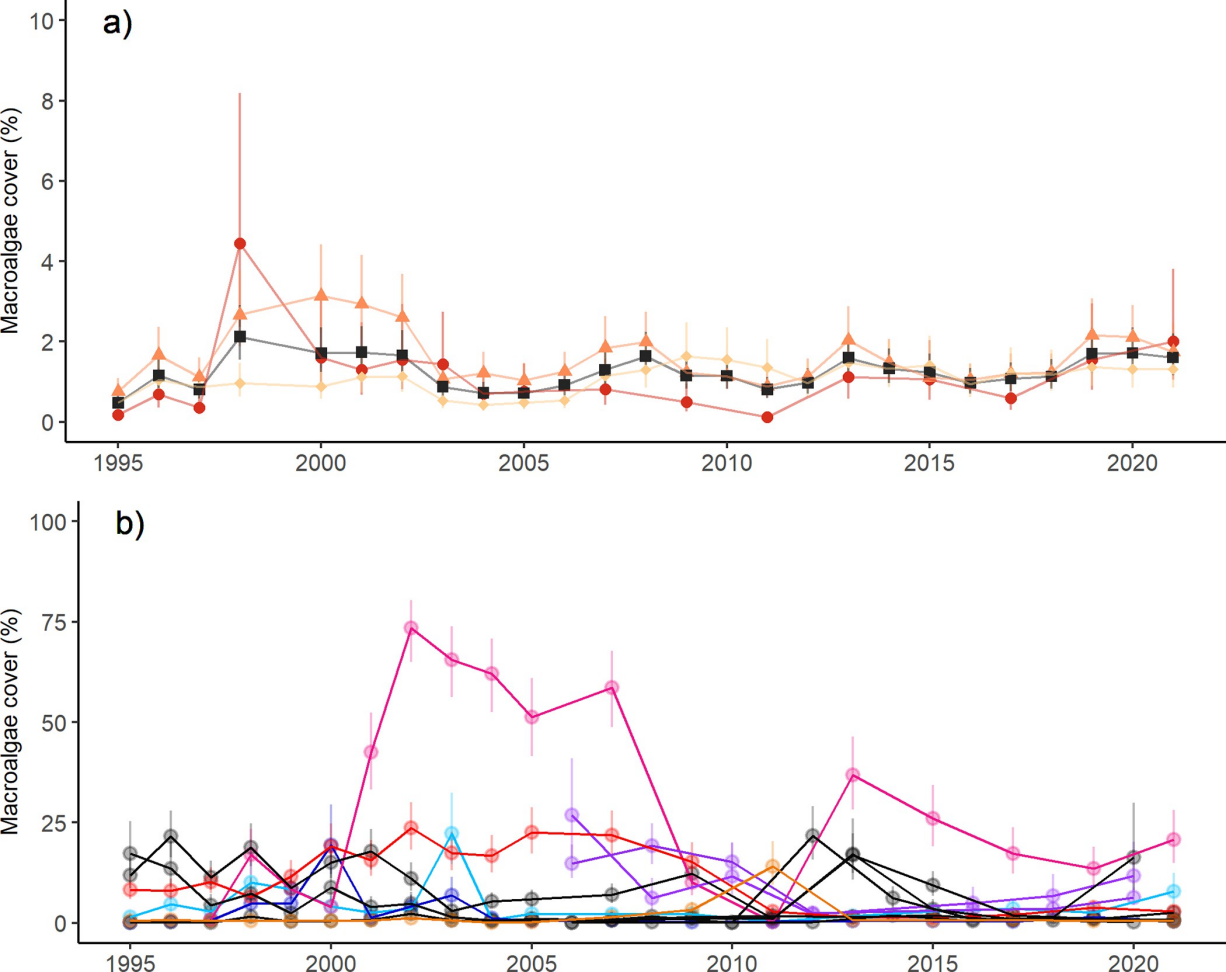
Temporal trend in total macroalgal cover (%) in the Great Barrier Reef between 1995 and 2021. Data are modelled hierarchical posterior distribution means and associated 95% Credible Intervals (CIs) from Bayesian hierarchical linear mixed models. (a) GBR-wide trend (black), as well as the trends for the inshore (red), mid-shelf (orange) and outer-shelf reefs (light orange) reefs. (b) Trends for the 12 reefs with the highest modelled MA values in anyone survey year. Pink: Havannah Reef (Townsville inshore); Light blue: Green Island Reef (Cairns inshore); Dark blue: Low Isles Reef (Cairns inshore); Red: Davies Reef (Townsville mid-shelf); Purple: Central mid-shelf reefs; Grey: Southern mid-shelf reefs; Light orange: Hyde Reef (Whitsunday outer-shelf).

The long-term monitoring data showed that MA averaged 1.3% at the 6–9 m deep LTMP sites at up to 93 reefs (averaged modelled means across all time points) and did not systematically change over time (Fig 7A). MA was most dynamic on the 11 inshore reefs represented. Inshore, annual mean MA values were highest in the surveys following mass bleaching events in 1998, 2000 and 2021 (4.4% (2.4–8.2% CIs), 1.6% (0.8–3.0% CIs) and 2% (1.1–3.8% CIs) respectively), and lowest after severe tropical cyclone Yasi in 2011 with 0.1% (0.06–0.2% CIs). On the mid-shelf, MA was highest between 1998 and 2002, ranging from 2.6% (2002; 1.8–3.7% CIs)– 3.1% (2.2–4.4% CIs), driven particularly by *Halimeda* and ’Algae other’, particularly at John Brewer, Rib Reef, 21–529, Davies Reef and Reef 20–104. In contrast, on outer shelf reefs MA was consistently low. Since 2012, MA cover has been similar for LTMP sites on all shelf positions. MA barely exceeded 2% in any shelf position since 2003. Temporal changes of most of the 93 individual reefs displayed no clear trends, with a few noteworthy exceptions (Fig 7B). Firstly, on the inshore reef Havannah Island, MA increased dramatically in 2001 and 2002 following multiple disturbances (Cyclone Tessi, a crown-of-thorns starfish outbreak and 2002 mass bleaching), and these levels only slowly waned over 20 years, interrupted by a brief drop in 2009 to 2011, as previously described [5]. This reef had the highest long-term mean MA (~30%), primarily *Lobophora* spp., with other Phaeophyceae present. MA also spiked at the inshore reefs Green Island Reef in 2003, and Low Isles in 2000, both following bleaching events. Second, while most mid-shelf and all outer-shelf reefs had low MA throughout, two southern (Reef 20–104, Reef 21–529) and three central (Davies, Farquharson, Centipede) mid-shelf reefs had periods with relatively high MA. For example, the Townsville mid-shelf reefs Davies and Reef 21–529 had persistently higher MA until 2011, mostly represented by *Halimeda* spp. (Fig 1G). Cyclone Yasi in 2011 was likely responsible for clearing out the well-established beds of *Halimeda* spp.

## Discussion

Our large-scale and long-term data from the GBR revealed the relative and cumulative contributions of the main spatial and environmental predictors and temporal changes in total macroalgal cover (MA). Systematic data of macroalgae are almost non-existent prior to ~1980, hence ‘pristine’ MA values are largely unknown and possibly under-estimated [17]. In the absence of baseline knowledge, extensive empirical contemporary data on spatial gradients and temporal dynamics in MA are indispensable to inform reef management.

Several caveats need to be considered when interpreting the results. First, the surveys were conducted at the level of ‘total macroalgal cover’, ignoring the many profound differences between functional and morphological groups (e.g., between fleshy algae and calcareous green and red macroalgae). Taxonomic inventories are far more sensitive indicators of environmental changes [51], however the taxonomic expertise to assess the megadiverse macroalgal assemblages on tropical reefs at a finer taxonomic level is often missing, making MA a more widely available indicator of change. Our data demonstrate that even at this broad level valuable information on the spatial distribution of MA across the GBR was evident. In addition, given the generally low cover of MA at most locations any important increases in MA will most likely be detectable that may then prompt more detailed assessment of the taxa involved. Second, local data for herbivore abundances and feeding rates were not available at relevant scales. Third, the one-off surveys were conducted between 1997 and 2008 when GBR-wide hard coral cover averaged 20–25% [37], and were spread across all seasons due to ship-time availability. The observed spatial patterns, albeit strong and consistent with biological knowledge, might have been even stronger through the inclusion of herbivore data and had the surveys been seasonally constrained [37], Last, the LTMP data comprise only few inshore reefs and transects are at 6–9 m depth, hence they do not represent those shallow inshore habitats where MA are most prolific and potentially most dynamic.

### Responses of macroalgae to spatial and environmental factors

Our data showed that habitats with particularly high MA are shallow inshore reefs with low hard coral cover, especially towards the southern end of the GBR. The increase in MA towards higher latitudes documented here has not been previously reported for the GBR. Survey data are unsuitable to confirm the ultimate causes of this latitudinal increase, since latitudinal gradients are confounded with changes in sea surface temperature, irradiance, tidal range, herbivore metabolic rates, carbonate chemistry, and other factors not considered here. However our finding agrees with other studies that show that MA continues to increase south of the GBR in subtropical waters [52], with communities eventually converting into temperate kelp forests. MA has also been reported to increase with latitude in the Caribbean, the Red Sea, the Hawaii’an Islands and Western Australia, typically attributed to declining sea surface temperature and increasing seawater chlorophyll and nutrient concentrations [53–57]. Importantly, reduced grazing pressure can also contribute to latitudinal changes, as predominant herbivores shift from fish to urchins [58] and common herbivores show lower bite rates hence lower *Sargassum* removal in a southern compared to a northern GBR region [59]. High MA biomass in coastal and shallow waters are well described [13, 16, 27], and linked to nutrient- and light stimulated production inshore and in shallow water, as well as to cross-shelf gradients in herbivory [60]. However, due to complex behavioral, dietary and metabolic filters, herbivore abundances may not always be related to grazing pressure and hence to macroalgal abundances [59].

The likelihood of MA dominance also increased with increasing tidal range and wave exposure, and with declining Secchi depth, aragonite saturation state, and sea surface temperature. The responses of MA to these factors were strong, and in accordance with known or expected biological relationships. For example, studies from Palau, Japan and the GBR concluded that waves and depth were among the most important drivers for coral reef associated macroalgae and *Sargassum* in particular [4, 16, 61]. Waves and strong currents can dislodge more ephemeral algae with weak attachment points, and can deter herbivores with low mobility, especially invertebrates [62]. However waves and currents increase nutrient and gas exchange on algal thalli, and remove [62] sediment deposits on macroalgae thalli, thus aiding gas exchange, and improving photosynthesis, growth and biomass [63].

Our data also confirmed the important role of water clarity, especially Secchi depth and PAR as predictors for MA [16, 27]. Secchi depth serves as proxy for concentrations of suspended particulate organic and inorganic matter, which some algal taxa including *Sargassum* demineralize after deposition on their fronds, potentially via epiphytic microbes [64]. On inshore reefs of the central and northern GBR, MA are 50% higher on turbid reefs than on otherwise comparable reefs with lower turbidity [33, 65]. Hence, MA has been used in support of water quality guidelines for turbidity and chlorophyll concentrations in the GBR [27, 28]. The weak prediction power for MA by eReefs-simulated long-term mean dissolved inorganic nitrogen and chlorophyll (as a proxy for nutrient availability) may be partially attributable to eReefs model limitations (e.g., strong simulated influences of wet seasons and offshore upwelling on DIN, plumes dynamics resolved only for larger river discharges [39], and the ephemeral nature of DIN). Published evidence for nutrient limitations in macroalgae shows strong associations in some studies [16], and weak or no relationships in others [61, 66]. Experimental studies show productivity increases lasting for weeks after nutrient pulses [67]. Field data also show that MA tends to increase on reefs with high nutrients either from human activities [68, 69] or upwelling [53], and MA decreased after sewage diversion [70]. Also, some of the benefits of higher nutrients in highly productive waters may be offset by light limitation due to PAR absorption from turbidity. Additionally, nutrient uptake is mass transfer limited and hence not only dependent on concentrations but also on currents and waves disrupting the boundary layer around tissues [62, 63]. All these factors show the complexity of inshore coral systems exposed to cumulative impacts from multiple pressures. Furthermore, sedimentation may depress algal food quality and rates of herbivory [71], hence water quality may benefit macroalgae not only directly through nutrient provisioning, but also indirectly through the release of grazing pressure, reconfirming that a dichotomous bottom-up or top-down model of macroalgal control is too simplistic to be ecologically useful [72]. All these factors demonstrate the complexity of inshore coral systems exposed to cumulative impacts from multiple pressures.

### Balance between macroalgae and hard corals, temporal trends, and management implications

Many studies have shown negative relationships between algal biomass and coral recruitment, and concluded that reef management actions to target lower algal biomass would aid reef recovery [73, 74]. Our data showed that on the GBR, the relationship between MA and hard coral cover was subject to strong spatial and environmental constraints, modulating the role of space competition between these two groups. Macroalgal dominance was restricted to wave-exposed shallow inshore reef flats, crests and upper slopes with low hard coral cover, whereas other reef habitats with low hard coral cover did not shift to MA dominance. Hence deeper reef slopes, mid-shelf and outer-shelf reef habitats were typically vacated by macrobenthos after disturbances, and although some showed temporary increases in MA, only one showed a persistent shift to MA dominance. Indeed, on many Indo-Pacific and also on some Caribbean reefs, space vacated after coral mortality is typically occupied by turf and coralline algae while macroalgae remain sparse (Fig 1H), with fast-growing foliose and tabulate corals eventually outcompeting and replacing these algal communities [5, 20, 32, 75].

Our 26 years of LTMP data showed no consistent temporal trends, despite the strong temporal trends previously reported for hard corals at the same reefs [37, 76]. MA was temporally most dynamic on inshore and mid-shelf reefs between 1998 and 2003, but events that caused coral mortality typically led to only brief, if any, spikes in MA. Havannah Reef was the only exception of the 93 reefs surveyed, where macroalgal dominance built up within a period of two years and waned only very gradually over two decades. Overall, the data largely show decadal stability in MA, notwithstanding the important caveat that no shallow sites and only few inshore reefs are represented in the LTMP data.

Ecological models validated by empirical data are needed to assess the cumulative net effects of predicted global and regional changes on reefs, including progressive warming, seawater acidification, greater fluctuations in terrestrial runoff, and intensifying tropical cyclones. Our data of MA and their drivers may help improve ecological models, further supporting predictions about the balance between hard corals and macroalgae on future coral reefs. We showed that MA was well predicted by seawater aragonite saturation state (and other closely related carbonate chemistry parameters), despite MA’s vast physiological diversity, with contrasting carbon concentrating mechanisms and CO_2_ sensitive biogenic calcification [77, 78]. Exchanging space for time suggests that progressively increasing seawater CO_2_ from ocean acidification may contribute to promoting MA [66], while warming may be depressing MA, complicating predictions. Increasing bleaching and cyclone intensities will most profoundly affect the GBR, with the LTMP data showing that tropical cyclones can lead to drops or spikes in MA.

The use of MA as one of several variables in a reef health indicator must consider underlying distribution patterns. On inshore reefs, the predictors latitude, distance across the shelf, depth, seawater chemistry and hydrodynamics should be considered to set location-specific MA thresholds. This would facilitate the detection of change while setting realistic expectations for the level of MA at a location. Observed changes should provide an indicator sensitive to potential shifts in top-down (herbivory) or bottom-up (seawater chemistry) controls, with observed changes in MA to be followed up at finer taxonomic resolution of the algal taxa involved.

To date, direct MA management options are limited. Among reactive MA management options, controlling sediment and nutrient runoff from agriculture and populated areas remains a top priority, benefiting a wide range of ecological processes on inshore reefs including mitigating eutrophication, sedimentation and non-atmospheric human-induced coastal acidification, and herbivory [66, 71, 79, 80]. Many studies have also confirmed the importance of maintaining balanced trophic levels in reef communities, including herbivorous fishes and their predators [73]. Herbivores are not typically fished on the GBR, but other constraints on their abundances such as turbidity or reef habitat complexity could be considered as additional restoration solution [30, 71]. Furthermore, macroalgal removal through manual or mechanical ‘weeding’ on small local scales and/or the targeted release of large herbivorous invertebrates is now increasingly being discussed or trialed [25, 29, 30]. To date their success is still limited, due to being small scale, labor intensive and transitory [25, 29]. About 40% of inshore reefs surveyed had one or more sites with ≥ 50% MA, however our data show that hard coral cover is strongly negatively related to MA only at shallow inshore sites, especially on windward reef flats and crests, and most often in the southern and central GBR. MA management planning could predominantly target these specific subsets of habitats. Should physical removal of macroalgae become a reef restoration choice [29, 31, 74], it would benefit corals mostly on shallow inshore reefs, which are far easier to access than deeper or offshore sites where low hard coral cover was not associated with macroalgal dominance. As direct MA management options are limited and costly, reef management considerations will benefit from our large-scale and long-term empirical MA distribution data, with certain types of southern inshore reefs having the propensity to establish macroalgal forests that are habitat for diverse assemblages of MA associated organisms [16].

## Supporting information

S1 File Supporting information–contains all the supporting figures, and supporting S1-S9 Tables. (DOCX)

S1 Table Fixed effects for modelled temporal trends of macroalgae cover on individual reefs are provided as separate.csv file due to the large size. Summary parameters are on the logit scale.
(CSV)

S2 Table Hyperparameters for modelled temporal trends of macroalgae cover on individual reefs are provided as separate.csv file due to the large size. Summary parameters are on the logit scale.
(CSV)
