# Supplementary material for: Macroalgal cover on coral reefs: Spatial and environmental predictors, and decadal trends in the Great Barrier Reef
Source: PLoS One. 2023 Jan 20;18(1):e0279699. doi: 10.1371/journal.pone.0279699 (PMC9858843; doi:10.1371/journal.pone.0279699)

Supplementary Materials

**Table S1:** Predictor variables used in the spatial and geomorphological (S, s) or the physical and biochemical environmental (E, e) aggregated boosted regression trees analyses, their units and definitions. Their retention in the final models, following cross-validation to minimize the prediction error, is indicated by upper case character under ‘Use’. For details of the definitions and calculations of the eReef model estimates see ^43^, ^61^, and ^62^.

| **Variable** | **Unit** | **Definition** | **Use** |
| --- | --- | --- | --- |
| Latitude | °S |  | S |
| Longitude | °E |  | s |
| *across* | n/a | Proportional distance across the continental shelf (0 = coast, 1 = outer edge of continental shelf) | S |
| *along* | n/a | Proportional distance along the continental shelf (0 = south, 1 = north) | s |
| Shelf position | (categorical) | Three cross-shelf categories: Inshore, mid-shelf, outer-shelf reef | s |
| Reef type | (categorical) | Three geomorphology categories: Coastal fringing, island fringing, and platform reef | s |
| Habitat type (location) | (categorical) | Four within-reef categories: windward front, leeward back, flank, lagoon site | s |
| Depth | m | Reef flat: 1 m  Reef crest: 2 m (3 - 1 m)  Upper slope: 5 m (8 - 3 m)  Mid slope: 10 m (13 - 8 m)  Deep slope: 15 m (18 - 13 m) (where available) | S |
| Season | (categorical) | Date of survey. Four 3-months blocks (1: Nov – Jan, 2: Feb – Apr, 3: May – Jul, 4: Aug – Oct) | s |
| Tidal range | m | eReefs: Long-term mean | E |
| Seawater temperature | °C | eReefs: Long-term mean at the depth of site | E |
| Salinity | (n/a) | eReefs: Long-term mean at the depth of site | e |
| Photosynthetically active radiation (PAR) | mol photons m^-2^ d^-1^ | eReefs: Long-term mean at the depth of site | E |
| Summer-winter difference in PAR | mol photons m^-2^ d^-1^ | eReefs: Long-term mean at the depth of site | e |
| PAR at 3.2 m depth | mol photons m^-2^ d^-1^ | eReefs: Long-term mean at 3.2 m depth (fixed depth relative to mean sea level corresponding to a model grid cell top surface) | e |
| Secchi depth | m | eReefs: Long-term mean.  Simulated Secchi depth is a measure of water transparency, calculated from the integral of attenuation of light at 488 nm | E |
| Turbidity | NTU | eReefs: Long-term mean, simulated using backscattering at 490 nm | e |
| Light attenuation coefficient (KD) | m^-1^ | eReefs: Long-term mean, coefficient, calculated from integral of attenuation of light at 490 nm | e |
| Suspended fine sediment | mg m^-3^ | eReefs: Long-term mean at the depth of site. Small (30 µm) mineral and carbonate mud particles that enter the domain from rivers | e |
| Dissolved inorganic nitrogen (DIN) | µmol L^-1^ | eReefs: Long-term mean at the depth of site | e |
| Total chlorophyll | mg m^-3^ | eReefs: Long-term mean at the depth of site. Chlorophyll a (mg m ^-3^), as the sum of small and large phytoplankton cells including *Trichodesmium* | e |
| Dissolved inorganic carbon | µmol kg^-1^ seawater | eReefs: Long-term mean at the depth of site. (DIC = CO_2_ + HCO_3_^-^ + CO_3_^2-^) | e |
| Total alkalinity (A_T_) | µmol kg^-1^ seawater | eReefs: Long-term mean at the depth of site | E |
| Aragonite saturation state (Ω_ar_) | (n/a) | Calculated from eReefs simulated A_T_, DIC, salinity and temperature at depth of site | E |
| Calcite saturation state (Ω_ca_) | (n/a) | Calculated from eReefs simulated A_T_, DIC, salinity and temperature at depth of site | e |
| pH | (n/a) | Calculated from eReefs simulated A_T_, DIC, salinity and temperature at depth of site | e |
| Partial pressure of CO_2_ (*p*CO_2_) | µatm | Calculated from eReefs simulated A_T_, DIC, salinity and temperature at depth of site | e |
| Sediment deposits | 4-point scale | Visual estimate of the amount of sediment deposited on the reef substratum in site (0 = none; 3 = too much to resuspend by fanning with slate) | e |
| Wave exposure | 5-point scale | Visual estimate of the amount of sediment deposited on the reef substratum in site (0 = none; 4 = extreme) | E |
| Slope angle | ° | Visual estimate across site (0° = horizontal, 90° = vertical) | e |

**Fig S1:** The probability of the presence of macroalgae at five depths and across three shelf positions from Rapid Ecological Assessment (REA) surveys.


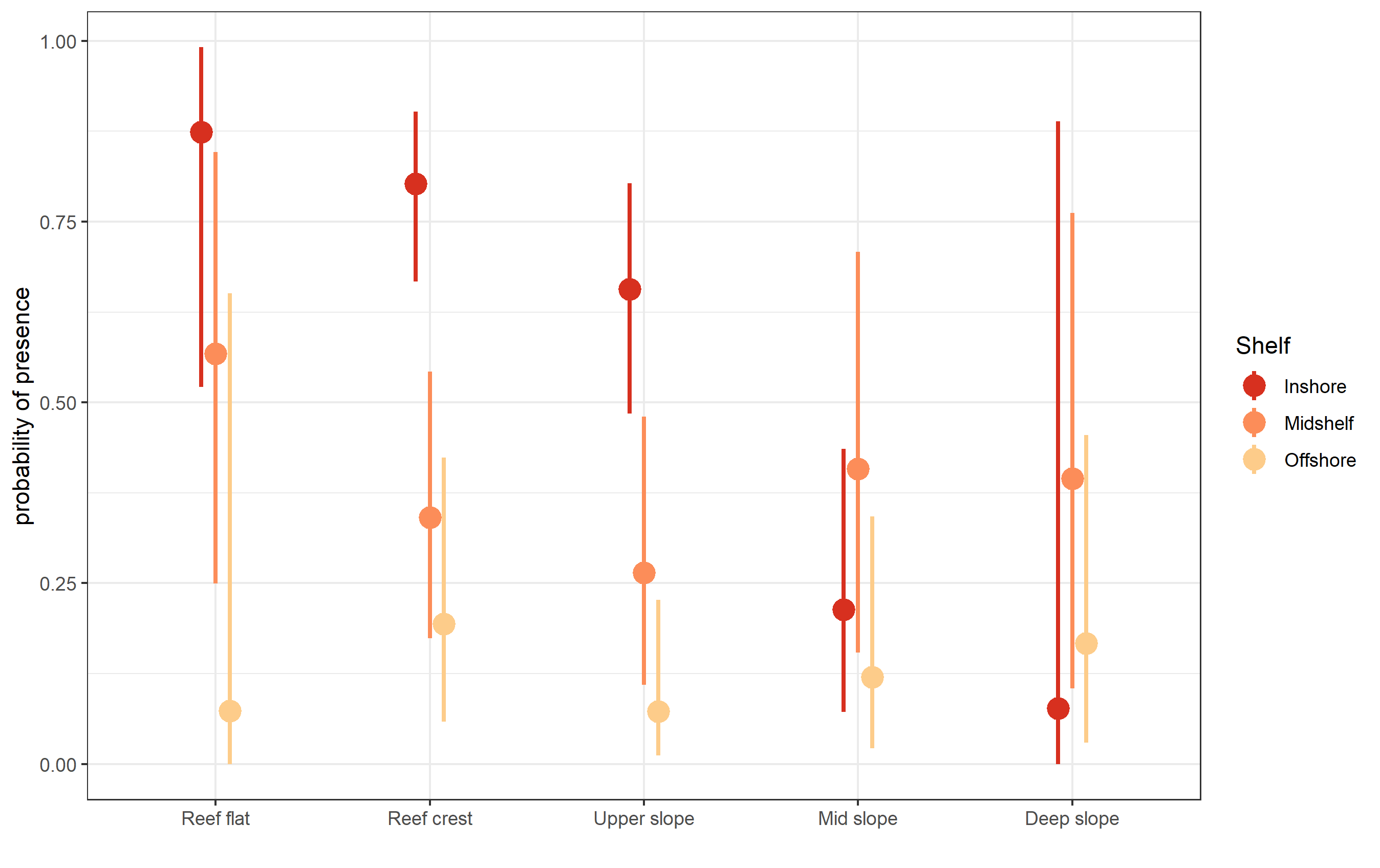


**Table S2:** Fixed effects for the modelled probability of macroalgae presence, given the population effects of hard coral cover, shelf position and depth, using data from Rapid Ecological Assessment (REA) surveys. Summary parameters are on the logit scale. Fixed effects terminology include: hc (Hard Coral), M (mid-shelf), O (Outer-shelf).

|  | mean | sd | 0.025quant | 0.5quant | 0.975quant | kld |
| --- | --- | --- | --- | --- | --- | --- |
| (Intercept) | 1.6015 | 0.4281 | 0.7794 | 1.5951 | 2.4596 | 2.06E-07 |
| hc | 0.0039 | 0.0111 | -0.0173 | 0.0037 | 0.0261 | 5.29E-08 |
| depth5 | -0.7506 | 0.4538 | -1.6485 | -0.7479 | 0.1319 | 6.15E-07 |
| depth10 | -1.7151 | 0.4809 | -2.6730 | -1.7101 | -0.7861 | 2.26E-07 |
| depth15 | -2.0546 | 0.7420 | -3.4764 | -2.0669 | -0.5633 | 4.16E-07 |
| depth1 | 0.8329 | 0.5386 | -0.2160 | 0.8302 | 1.8969 | 9.11E-07 |
| ShelfM | -1.4903 | 0.8116 | -3.0913 | -1.4873 | 0.0942 | 7.88E-07 |
| ShelfO | -4.0331 | 1.2009 | -6.4606 | -4.0079 | -1.7482 | 1.02E-07 |
| hc:depth5 | -0.0031 | 0.0143 | -0.0313 | -0.0030 | 0.0247 | 6.89E-07 |
| hc:depth10 | -0.0377 | 0.0189 | -0.0766 | -0.0371 | -0.0024 | 1.74E-06 |
| hc:depth15 | -0.1804 | 0.1165 | -0.4423 | -0.1671 | 0.0120 | 7.92E-06 |
| hc:depth1 | 0.0062 | 0.0327 | -0.0523 | 0.0040 | 0.0763 | 5.81E-06 |
| hc:shelfM | -0.0265 | 0.0186 | -0.0637 | -0.0263 | 0.0094 | 2.66E-08 |
| hc:shelfO | 0.0019 | 0.0264 | -0.0505 | 0.0021 | 0.0532 | 3.24E-07 |
| depth5:shelfM | -0.2791 | 0.9068 | -2.0680 | -0.2755 | 1.4896 | 8.07E-07 |
| depth10:shelfM | 0.5663 | 0.9230 | -1.2518 | 0.5689 | 2.3693 | 8.26E-07 |
| depth15:shelfM | 1.4984 | 1.1543 | -0.7861 | 1.5054 | 3.7431 | 2.93E-07 |
| depth1:shelfM | 0.6002 | 1.0869 | -1.5177 | 0.5951 | 2.7466 | 6.22E-07 |
| depth5:shelfO | 0.9275 | 1.4200 | -1.8578 | 0.9274 | 3.7131 | 2.22E-07 |
| depth10:shelfO | 0.0065 | 1.4510 | -2.8693 | 0.0167 | 2.8239 | 3.77E-07 |
| depth15:shelfO | 1.6372 | 1.5693 | -1.4459 | 1.6388 | 4.7112 | 1.01E-06 |
| depth1:shelfO | 0.8641 | 2.7851 | -4.3993 | 0.7920 | 6.5368 | 1.22E-06 |
| hc:depth5:shelfM | 0.0166 | 0.0257 | -0.0336 | 0.0165 | 0.0671 | 8.63E-07 |
| hc:depth10:shelfM | 0.0725 | 0.0314 | 0.0122 | 0.0721 | 0.1355 | 7.01E-09 |
| hc:depth15:shelfM | 0.2018 | 0.1206 | 0.0011 | 0.1885 | 0.4716 | 7.79E-06 |
| hc:depth1:shelfM | -0.0149 | 0.0439 | -0.1047 | -0.0136 | 0.0677 | 1.73E-06 |
| hc:depth5:shelfO | -0.0344 | 0.0381 | -0.1121 | -0.0334 | 0.0375 | 1.38E-06 |
| hc:depth10:shelfO | 0.0602 | 0.0406 | -0.0195 | 0.0602 | 0.1400 | 6.69E-07 |
| hc:depth15:shelfO | 0.1880 | 0.1227 | -0.0171 | 0.1748 | 0.4620 | 7.71E-06 |
| hc:depth1:shelfO | -0.1353 | 0.1202 | -0.3873 | -0.1297 | 0.0850 | 4.04E-06 |

**Table S3:** Hyperparameters for the modelled probability of macroalgae presence, given the population effects of hard coral cover, shelf position and depth, using data from Rapid Ecological Assessment (REA) surveys. Summary parameters are on the logit scale.

|  | mean | sd | 0.025quant | 0.5quant | 0.975quant |
| --- | --- | --- | --- | --- | --- |
| Precision for reef | 0.4053 | 0.1196 | 0.2291 | 0.3856 | 0.6943 |
| Precision for site | 0.3908 | 0.1085 | 0.2259 | 0.3745 | 0.6483 |
| Precision for transect | 22954.7386 | 24004.9411 | 1694.8358 | 15808.5533 | 86221.1335 |

**Fig S2:** DHARMA residual diagnostics for the modelled probability of macroalgae presence, given the population effects of hard coral cover, shelf position and depth, using data from Rapid Ecological Assessment (REA) surveys.
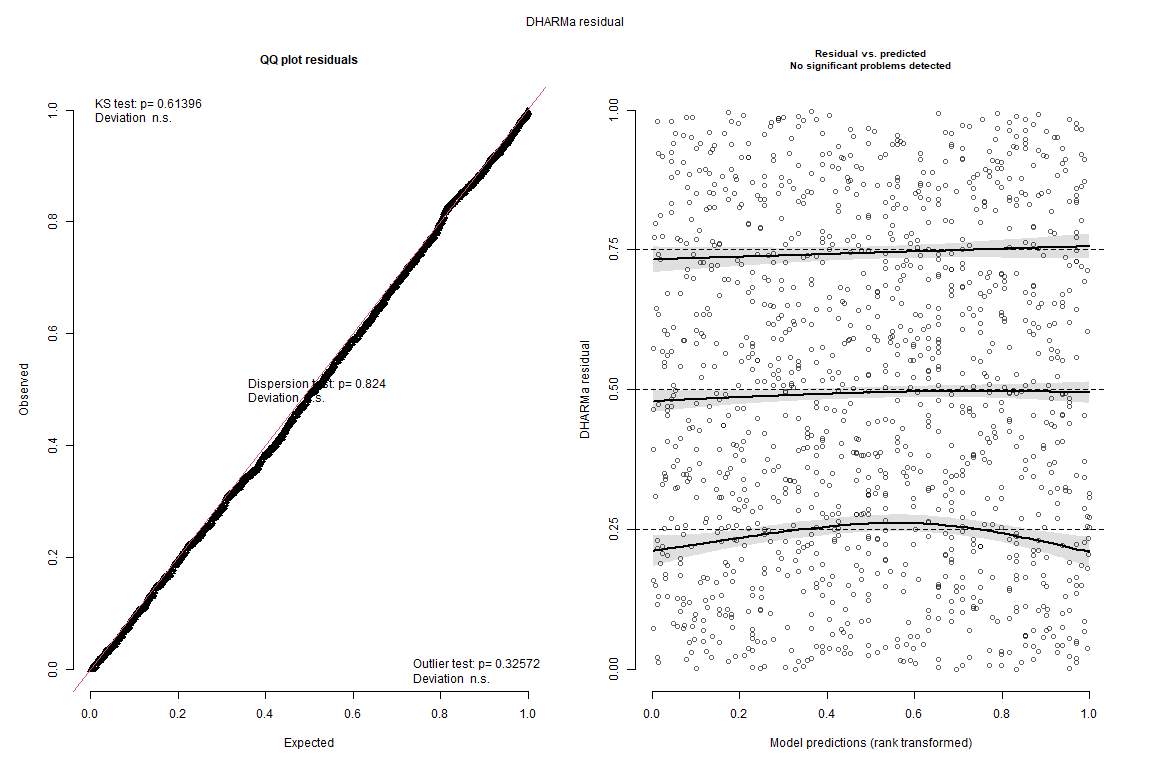


**Table S4:** Fixed effects for the modelled abundance of macroalgae, given the population effects of hard coral cover, shelf position and depth, using data from Rapid Ecological Assessment (REA) surveys. Summary parameters are on the logit scale. Fixed effects terminology include: hc (Hard Coral), M (mid-shelf), O (Outer-shelf).

|  | mean | sd | 0.025quant | 0.5quant | 0.975quant | kld |
| --- | --- | --- | --- | --- | --- | --- |
| (Intercept) | -0.4939 | 0.1456 | -0.7802 | -0.4937 | -0.2089 | 2.21E-07 |
| hc | -0.0255 | 0.0039 | -0.0332 | -0.0254 | -0.0180 | 1.22E-07 |
| depth5 | -0.7192 | 0.1533 | -1.0203 | -0.7190 | -0.4188 | 9.54E-07 |
| depth10 | -1.5419 | 0.1967 | -1.9288 | -1.5416 | -1.1570 | 4.24E-07 |
| depth15 | -1.8463 | 0.5007 | -2.8544 | -1.8374 | -0.8893 | 5.09E-07 |
| depth1 | 0.5458 | 0.1675 | 0.2171 | 0.5458 | 0.8741 | 9.70E-07 |
| shelfM | -1.3659 | 0.3922 | -2.1475 | -1.3616 | -0.6085 | 2.29E-08 |
| shelfO | -1.9691 | 0.7746 | -3.5624 | -1.9434 | -0.5204 | 2.28E-06 |
| hc:depth5 | 0.0045 | 0.0054 | -0.0061 | 0.0045 | 0.0150 | 7.71E-07 |
| hc:depth10 | 0.0230 | 0.0097 | 0.0029 | 0.0233 | 0.0410 | 2.92E-06 |
| hc:depth15 | 0.0326 | 0.1169 | -0.2151 | 0.0391 | 0.2440 | 5.04E-06 |
| hc:depth1 | -0.0344 | 0.0095 | -0.0535 | -0.0343 | -0.0162 | 3.35E-07 |
| hc:shelfM | 0.0175 | 0.0101 | -0.0027 | 0.0176 | 0.0371 | 1.06E-07 |
| hc:shelfO | 0.0199 | 0.0184 | -0.0162 | 0.0199 | 0.0560 | 3.93E-07 |
| depth5:shelfM | 0.2361 | 0.4477 | -0.6368 | 0.2342 | 1.1198 | 8.64E-07 |
| depth10:shelfM | 0.5878 | 0.4700 | -0.3293 | 0.5861 | 1.5145 | 8.45E-07 |
| depth15:shelfM | 1.3463 | 0.7646 | -0.1540 | 1.3466 | 2.8450 | 5.07E-08 |
| depth1:shelfM | -0.2779 | 0.4722 | -1.1970 | -0.2804 | 0.6554 | 5.97E-07 |
| depth5:shelfO | 1.0712 | 0.9281 | -0.7140 | 1.0587 | 2.9276 | 2.66E-08 |
| depth10:shelfO | 1.1424 | 0.9887 | -0.7694 | 1.1327 | 3.1092 | 5.50E-08 |
| depth15:shelfO | 2.5817 | 1.1756 | 0.2613 | 2.5869 | 4.8725 | 7.26E-07 |
| depth1:shelfO | 0.5538 | 1.8342 | -3.1587 | 0.5935 | 4.0412 | 6.59E-07 |
| hc:depth5:shelfM | 0.0036 | 0.0135 | -0.0231 | 0.0037 | 0.0300 | 8.76E-07 |
| hc:depth10:shelfM | -0.0040 | 0.0168 | -0.0368 | -0.0041 | 0.0290 | 7.60E-07 |
| hc:depth15:shelfM | -0.0177 | 0.1192 | -0.2342 | -0.0241 | 0.2341 | 4.80E-06 |
| hc:depth1:shelfM | 0.0190 | 0.0176 | -0.0157 | 0.0190 | 0.0532 | 7.31E-07 |
| hc:depth5:shelfO | -0.0102 | 0.0256 | -0.0621 | -0.0096 | 0.0383 | 9.44E-07 |
| hc:depth10:shelfO | -0.0145 | 0.0270 | -0.0698 | -0.0137 | 0.0363 | 1.77E-06 |
| hc:depth15:shelfO | -0.0551 | 0.1223 | -0.2781 | -0.0613 | 0.2022 | 4.48E-06 |
| hc:depth1:shelfO | -0.0125 | 0.0804 | -0.1725 | -0.0117 | 0.1431 | 5.10E-08 |

**Table S5:** Hyperparameters for the modelled abundance of macroalgae, given the population effects of hard coral cover, shelf position and depth, using data from Rapid Ecological Assessment (REA) surveys. Summary parameters are on the logit scale.

|  | mean | sd | 0.025quant | 0.5quant | 0.975quant |
| --- | --- | --- | --- | --- | --- |
| precision parameter for the beta observations | 7.2276 | 0.6616 | 5.9872 | 7.2078 | 8.5892 |
| Precision for reef | 1.7294 | 0.3925 | 1.0806 | 1.6884 | 2.6166 |
| Precision for site | 3.8936 | 1.2293 | 2.0704 | 3.6959 | 6.8492 |
| Precision for transect | 23306.5973 | 12087.2811 | 8274.0364 | 20573.5717 | 54168.4493 |

**Fig S3:** DHARMA residual diagnostics for the modelled abundance of macroalgae, given the population effects of hard coral cover, shelf position and depth, using data from Rapid Ecological Assessment (REA) surveys.
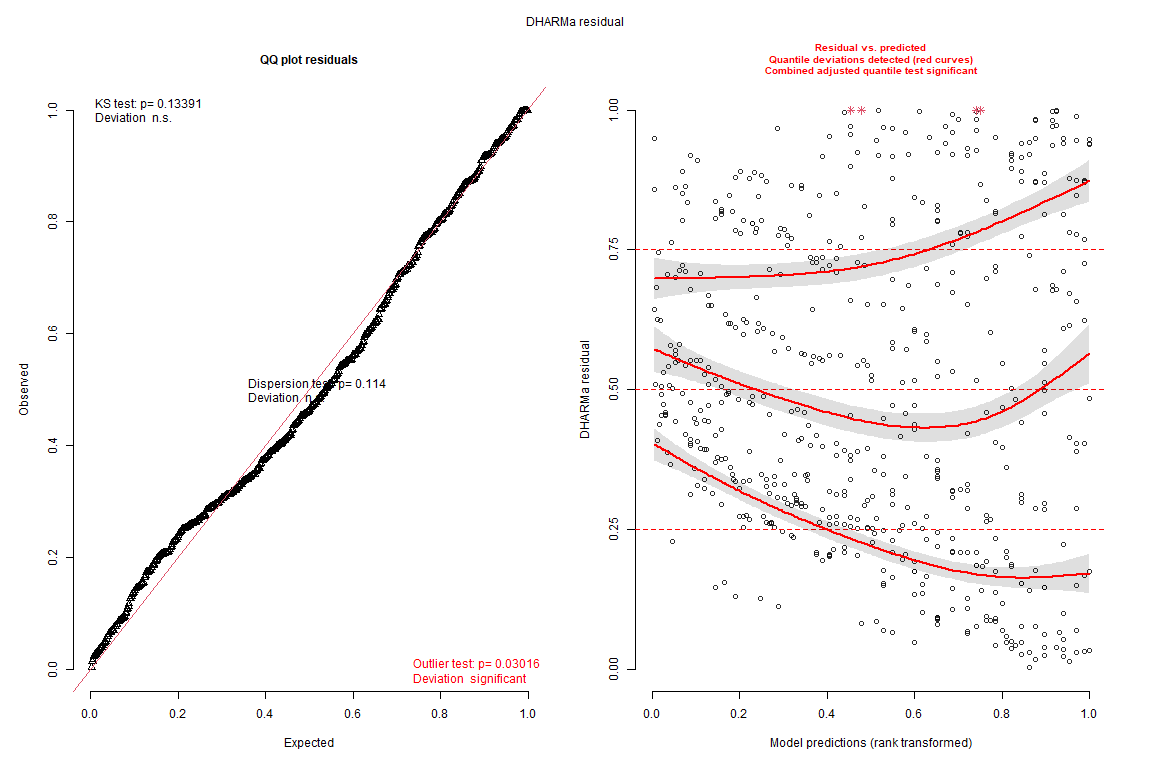


**Table S6:** Fixed effects for modelled temporal trends of macroalgae cover on the whole Great Barrier Reef (GBR). Summary parameters are on the logit scale.

|  | mean | sd | 0.025quant | 0.5quant | 0.975quant | kld |
| --- | --- | --- | --- | --- | --- | --- |
| (Intercept) | -5.3462 | 0.1699 | -5.6801 | -5.3461 | -5.0130 | 3.44E-07 |
| REPORT_YEAR1996 | 0.8981 | 0.0829 | 0.7358 | 0.8980 | 1.0608 | 8.60E-07 |
| REPORT_YEAR1997 | 0.5337 | 0.0844 | 0.3682 | 0.5336 | 0.6994 | 7.92E-07 |
| REPORT_YEAR1998 | 1.5148 | 0.0814 | 1.3554 | 1.5147 | 1.6747 | 9.15E-07 |
| REPORT_YEAR2000 | 1.2946 | 0.0826 | 1.1329 | 1.2946 | 1.4569 | 8.92E-07 |
| REPORT_YEAR2001 | 1.3091 | 0.0853 | 1.1419 | 1.3090 | 1.4767 | 8.60E-07 |
| REPORT_YEAR2002 | 1.2635 | 0.0851 | 1.0968 | 1.2634 | 1.4306 | 8.70E-07 |
| REPORT_YEAR2003 | 0.6116 | 0.0860 | 0.4431 | 0.6115 | 0.7803 | 7.96E-07 |
| REPORT_YEAR2004 | 0.4189 | 0.0851 | 0.2520 | 0.4188 | 0.5860 | 7.59E-07 |
| REPORT_YEAR2005 | 0.4152 | 0.0855 | 0.2477 | 0.4152 | 0.5831 | 7.72E-07 |
| REPORT_YEAR2006 | 0.6635 | 0.0942 | 0.4789 | 0.6634 | 0.8482 | 6.80E-07 |
| REPORT_YEAR2007 | 1.0141 | 0.0843 | 0.8490 | 1.0140 | 1.1796 | 8.54E-07 |
| REPORT_YEAR2008 | 1.2495 | 0.0913 | 1.0707 | 1.2495 | 1.4288 | 8.12E-07 |
| REPORT_YEAR2009 | 0.8934 | 0.0822 | 0.7323 | 0.8933 | 1.0549 | 8.74E-07 |
| REPORT_YEAR2010 | 0.8880 | 0.0900 | 0.7116 | 0.8879 | 1.0648 | 8.01E-07 |
| REPORT_YEAR2011 | 0.5387 | 0.0850 | 0.3722 | 0.5386 | 0.7055 | 8.26E-07 |
| REPORT_YEAR2012 | 0.7263 | 0.0897 | 0.5505 | 0.7263 | 0.9025 | 8.03E-07 |
| REPORT_YEAR2013 | 1.2247 | 0.0832 | 1.0617 | 1.2246 | 1.3881 | 8.72E-07 |
| REPORT_YEAR2014 | 1.0413 | 0.0914 | 0.8623 | 1.0412 | 1.2206 | 7.96E-07 |
| REPORT_YEAR2015 | 0.9622 | 0.0842 | 0.7973 | 0.9622 | 1.1277 | 8.64E-07 |
| REPORT_YEAR2016 | 0.7219 | 0.0924 | 0.5409 | 0.7218 | 0.9033 | 7.79E-07 |
| REPORT_YEAR2017 | 0.8248 | 0.0846 | 0.6591 | 0.8247 | 0.9909 | 8.50E-07 |
| REPORT_YEAR2018 | 0.8774 | 0.0909 | 0.6994 | 0.8774 | 1.0558 | 7.99E-07 |
| REPORT_YEAR2019 | 1.2882 | 0.0839 | 1.1240 | 1.2881 | 1.4529 | 8.74E-07 |
| REPORT_YEAR2020 | 1.2984 | 0.0912 | 1.1197 | 1.2983 | 1.4775 | 8.15E-07 |
| REPORT_YEAR2021 | 1.2295 | 0.0830 | 1.0669 | 1.2294 | 1.3926 | 8.89E-07 |

**Table S7:** Hyperparameters for modelled temporal trends of macroalgae cover on the whole Great Barrier Reef (GBR). Summary parameters are on the logit scale.

|  | mean | sd | 0.025quant | 0.5quant | 0.975quant |
| --- | --- | --- | --- | --- | --- |
| Precision for SHELF | 56.3501 | 9.0946 | 39.4305 | 56.1196 | 74.9460 |
| Precision for AIMS_REEF_NAME | 0.9927 | 0.1493 | 0.7393 | 0.9786 | 1.3255 |
| Precision for SITE_NO | 5.9011 | 0.9017 | 4.2093 | 5.8798 | 7.7382 |
| Precision for TRANSECT_NO | 47.7498 | 7.2854 | 34.5783 | 47.3742 | 63.1938 |
| Precision for month | 20.2763 | 2.1837 | 16.2449 | 20.1887 | 24.8310 |
| Precision for Obs | 0.7230 | 0.0129 | 0.6978 | 0.7229 | 0.7486 |

**Fig S4:** DHARMa residual diagnostics for modelled temporal trends of macroalgae cover on the whole GBR.


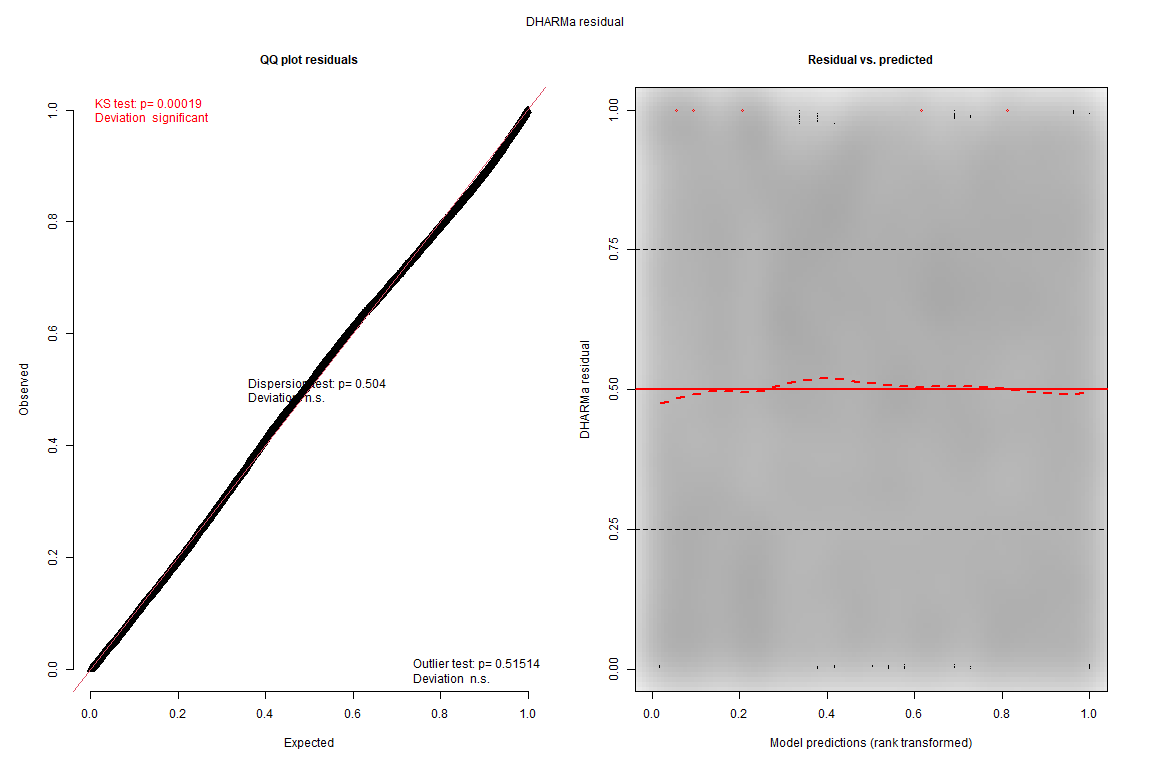


**Table S8:** Fixed effects for modelled temporal trends of macroalgae cover across 3 shelf positions. Summary parameters are on the logit scale. Fixed effects terminology include: M (mid-shelf), I (inshore).

|  | mean | sd | 0.025  quant | 0.5  quant | 0.975  quant | kld |
| --- | --- | --- | --- | --- | --- | --- |
| (Intercept) | -5.3328 | 0.2236 | -5.7718 | -5.3327 | -4.8944 | 5.28E-07 |
| REPORT_YEAR1996 | 0.7820 | 0.1256 | 0.5362 | 0.7819 | 1.0287 | 9.37E-07 |
| REPORT_YEAR1997 | 0.6007 | 0.1266 | 0.3528 | 0.6005 | 0.8493 | 9.11E-07 |
| REPORT_YEAR1998 | 0.6976 | 0.1265 | 0.4499 | 0.6975 | 0.9462 | 9.33E-07 |
| REPORT_YEAR2000 | 0.6086 | 0.1277 | 0.3586 | 0.6085 | 0.8594 | 9.09E-07 |
| REPORT_YEAR2001 | 0.8534 | 0.1282 | 0.6024 | 0.8533 | 1.1053 | 9.25E-07 |
| REPORT_YEAR2002 | 0.8662 | 0.1273 | 0.6169 | 0.8660 | 1.1163 | 9.41E-07 |
| REPORT_YEAR2003 | 0.1000 | 0.1317 | -0.1582 | 0.0999 | 0.3586 | 7.61E-07 |
| REPORT_YEAR2004 | -0.1125 | 0.1323 | -0.3720 | -0.1126 | 0.1471 | 6.14E-07 |
| REPORT_YEAR2005 | 0.0119 | 0.1317 | -0.2462 | 0.0118 | 0.2704 | 7.39E-07 |
| REPORT_YEAR2006 | 0.1134 | 0.1444 | -0.1698 | 0.1133 | 0.3965 | 1.24E-07 |
| REPORT_YEAR2007 | 0.8763 | 0.1264 | 0.6288 | 0.8761 | 1.1246 | 9.36E-07 |
| REPORT_YEAR2008 | 1.0019 | 0.1362 | 0.7352 | 1.0018 | 1.2694 | 8.82E-07 |
| REPORT_YEAR2009 | 1.2349 | 0.1231 | 0.9939 | 1.2347 | 1.4769 | 9.71E-07 |
| REPORT_YEAR2010 | 1.1827 | 0.1348 | 0.9187 | 1.1825 | 1.4474 | 8.94E-07 |
| REPORT_YEAR2011 | 1.0453 | 0.1252 | 0.8002 | 1.0451 | 1.2912 | 9.57E-07 |
| REPORT_YEAR2012 | 0.7049 | 0.1363 | 0.4380 | 0.7048 | 0.9724 | 8.63E-07 |
| REPORT_YEAR2013 | 1.1324 | 0.1255 | 0.8867 | 1.1322 | 1.3790 | 9.56E-07 |
| REPORT_YEAR2014 | 1.0254 | 0.1379 | 0.7552 | 1.0252 | 1.2962 | 8.73E-07 |
| REPORT_YEAR2015 | 1.0849 | 0.1253 | 0.8396 | 1.0848 | 1.3312 | 9.60E-07 |
| REPORT_YEAR2016 | 0.6750 | 0.1383 | 0.4040 | 0.6749 | 0.9466 | 8.31E-07 |
| REPORT_YEAR2017 | 0.9370 | 0.1269 | 0.6886 | 0.9368 | 1.1862 | 9.42E-07 |
| REPORT_YEAR2018 | 0.9021 | 0.1364 | 0.6350 | 0.9020 | 1.1700 | 8.73E-07 |
| REPORT_YEAR2019 | 1.0543 | 0.1273 | 0.8050 | 1.0542 | 1.3045 | 9.40E-07 |
| REPORT_YEAR2020 | 1.0152 | 0.1416 | 0.7378 | 1.0151 | 1.2931 | 7.81E-07 |
| REPORT_YEAR2021 | 1.0128 | 0.1265 | 0.7651 | 1.0126 | 1.2614 | 9.46E-07 |
| SHELFM | 0.4616 | 0.2742 | -0.0764 | 0.4616 | 0.9998 | 1.66E-07 |
| SHELFI | -0.9819 | 0.4126 | -1.7925 | -0.9816 | -0.1732 | 8.69E-07 |
| REPORT_YEAR1996:SHELFM | 0.0113 | 0.1735 | -0.3290 | 0.0113 | 0.3514 | 3.01E-07 |
| REPORT_YEAR1997:SHELFM | -0.2069 | 0.1763 | -0.5528 | -0.2069 | 0.1389 | 3.77E-07 |
| REPORT_YEAR1998:SHELFM | 0.5754 | 0.1719 | 0.2381 | 0.5754 | 0.9126 | 2.28E-07 |
| REPORT_YEAR2000:SHELFM | 0.8323 | 0.1728 | 0.4935 | 0.8323 | 1.1712 | 1.17E-07 |
| REPORT_YEAR2001:SHELFM | 0.5175 | 0.1735 | 0.1773 | 0.5175 | 0.8577 | 2.48E-07 |
| REPORT_YEAR2002:SHELFM | 0.3837 | 0.1713 | 0.0477 | 0.3837 | 0.7197 | 2.71E-07 |
| REPORT_YEAR2003:SHELFM | 0.2434 | 0.1778 | -0.1054 | 0.2434 | 0.5922 | 3.45E-08 |
| REPORT_YEAR2004:SHELFM | 0.5886 | 0.1793 | 0.2371 | 0.5886 | 0.9402 | 2.90E-07 |
| REPORT_YEAR2005:SHELFM | 0.2925 | 0.1789 | -0.0583 | 0.2925 | 0.6433 | 4.06E-08 |
| REPORT_YEAR2006:SHELFM | 0.3945 | 0.1901 | 0.0218 | 0.3945 | 0.7675 | 4.90E-07 |
| REPORT_YEAR2007:SHELFM | 0.0211 | 0.1742 | -0.3206 | 0.0211 | 0.3627 | 3.30E-07 |
| REPORT_YEAR2008:SHELFM | -0.0294 | 0.1831 | -0.3885 | -0.0294 | 0.3297 | 1.38E-07 |
| REPORT_YEAR2009:SHELFM | -0.7513 | 0.1724 | -1.0896 | -0.7512 | -0.4132 | 4.68E-07 |
| REPORT_YEAR2010:SHELFM | -0.7825 | 0.1829 | -1.1413 | -0.7825 | -0.4237 | 2.26E-07 |
| REPORT_YEAR2011:SHELFM | -0.8938 | 0.1737 | -1.2347 | -0.8938 | -0.5532 | 4.64E-07 |
| REPORT_YEAR2012:SHELFM | -0.3035 | 0.1840 | -0.6643 | -0.3035 | 0.0572 | 1.17E-07 |
| REPORT_YEAR2013:SHELFM | -0.1381 | 0.1720 | -0.4756 | -0.1381 | 0.1993 | 3.68E-07 |
| REPORT_YEAR2014:SHELFM | -0.3502 | 0.1854 | -0.7138 | -0.3502 | 0.0133 | 1.95E-07 |
| REPORT_YEAR2015:SHELFM | -0.7102 | 0.1731 | -1.0496 | -0.7101 | -0.3708 | 4.32E-07 |
| REPORT_YEAR2016:SHELFM | -0.3548 | 0.1845 | -0.7167 | -0.3548 | 0.0070 | 5.35E-09 |
| REPORT_YEAR2017:SHELFM | -0.4772 | 0.1725 | -0.8155 | -0.4772 | -0.1391 | 3.66E-07 |
| REPORT_YEAR2018:SHELFM | -0.4204 | 0.1841 | -0.7815 | -0.4204 | -0.0594 | 1.48E-07 |
| REPORT_YEAR2019:SHELFM | 0.0051 | 0.1738 | -0.3359 | 0.0051 | 0.3459 | 2.73E-07 |
| REPORT_YEAR2020:SHELFM | 0.0186 | 0.1864 | -0.3471 | 0.0185 | 0.3842 | 1.77E-07 |
| REPORT_YEAR2021:SHELFM | -0.1730 | 0.1728 | -0.5119 | -0.1730 | 0.1658 | 3.09E-07 |
| REPORT_YEAR1996:SHELFI | 0.5615 | 0.2458 | 0.0818 | 0.5607 | 1.0459 | 8.47E-07 |
| REPORT_YEAR1997:SHELFI | 0.0889 | 0.2478 | -0.3948 | 0.0881 | 0.5770 | 8.72E-07 |
| REPORT_YEAR1998:SHELFI | 2.5495 | 0.2342 | 2.0931 | 2.5485 | 3.0119 | 6.77E-07 |
| REPORT_YEAR2000:SHELFI | 1.5867 | 0.2378 | 1.1231 | 1.5857 | 2.0559 | 7.25E-07 |
| REPORT_YEAR2001:SHELFI | 1.1310 | 0.2393 | 0.6643 | 1.1301 | 1.6031 | 7.56E-07 |
| REPORT_YEAR2002:SHELFI | 1.2958 | 0.2383 | 0.8313 | 1.2949 | 1.7658 | 7.43E-07 |
| REPORT_YEAR2003:SHELFI | 1.9877 | 0.2409 | 1.5179 | 1.9868 | 2.4629 | 7.60E-07 |
| REPORT_YEAR2004:SHELFI | 1.4726 | 0.2467 | 0.9912 | 1.4718 | 1.9589 | 8.30E-07 |
| REPORT_YEAR2005:SHELFI | 1.4209 | 0.2443 | 0.9443 | 1.4200 | 1.9025 | 8.07E-07 |
| REPORT_YEAR2006:SHELFI | 0.0000 | 31.6225 | -62.0167 | 0.0000 | 62.0167 | 5.53E-11 |
| REPORT_YEAR2007:SHELFI | 0.6407 | 0.2420 | 0.1685 | 0.6398 | 1.1178 | 8.03E-07 |
| REPORT_YEAR2008:SHELFI | 0.0000 | 31.6225 | -62.0167 | 0.0000 | 62.0167 | 5.53E-11 |
| REPORT_YEAR2009:SHELFI | -0.2261 | 0.2432 | -0.7006 | -0.2269 | 0.2532 | 8.26E-07 |
| REPORT_YEAR2010:SHELFI | 0.0000 | 31.6225 | -62.0167 | 0.0000 | 62.0167 | 5.53E-11 |
| REPORT_YEAR2011:SHELFI | -1.4974 | 0.2629 | -2.0122 | -1.4977 | -0.9809 | 9.44E-07 |
| REPORT_YEAR2012:SHELFI | 0.0000 | 31.6225 | -62.0167 | 0.0000 | 62.0167 | 5.53E-11 |
| REPORT_YEAR2013:SHELFI | 0.6964 | 0.2391 | 0.2302 | 0.6955 | 1.1679 | 7.64E-07 |
| REPORT_YEAR2014:SHELFI | 0.0000 | 31.6225 | -62.0167 | 0.0000 | 62.0167 | 5.53E-11 |
| REPORT_YEAR2015:SHELFI | 0.6949 | 0.2421 | 0.2227 | 0.6940 | 1.1722 | 7.87E-07 |
| REPORT_YEAR2016:SHELFI | 0.0000 | 31.6225 | -62.0167 | 0.0000 | 62.0167 | 5.53E-11 |
| REPORT_YEAR2017:SHELFI | 0.2443 | 0.2460 | -0.2359 | 0.2435 | 0.7291 | 8.45E-07 |
| REPORT_YEAR2018:SHELFI | 0.0000 | 31.6225 | -62.0167 | 0.0000 | 62.0167 | 5.53E-11 |
| REPORT_YEAR2019:SHELFI | 1.1079 | 0.2419 | 0.6362 | 1.1069 | 1.5848 | 7.66E-07 |
| REPORT_YEAR2020:SHELFI | 0.0000 | 31.6225 | -62.0167 | 0.0000 | 62.0167 | 5.53E-11 |
| REPORT_YEAR2021:SHELFI | 1.4161 | 0.2393 | 0.9496 | 1.4151 | 1.8881 | 7.41E-07 |

**Table S9:** Hyperparameters for modelled temporal trends of macroalgae cover across 3 shelf positions. Summary parameters are on the logit scale.

|  | mean | sd | 0.025quant | 0.5quant | 0.975quant |
| --- | --- | --- | --- | --- | --- |
| Precision for AIMS_REEF_NAME | 0.8891 | 0.1571 | 0.5727 | 0.8976 | 1.1686 |
| Precision for SITE_NO | 5.9103 | 0.6373 | 4.7030 | 5.8994 | 7.1999 |
| Precision for TRANSECT_NO | 30.6764 | 6.9370 | 17.2397 | 30.8772 | 43.4791 |
| Precision for month | 17.9494 | 1.4143 | 15.4176 | 17.8592 | 20.9676 |
| Precision for Obs | 0.7761 | 0.0106 | 0.7545 | 0.7764 | 0.7960 |

**Fig S5:** DHARMa residual diagnostics for modelled temporal trends of macroalgae cover across shelf positions.


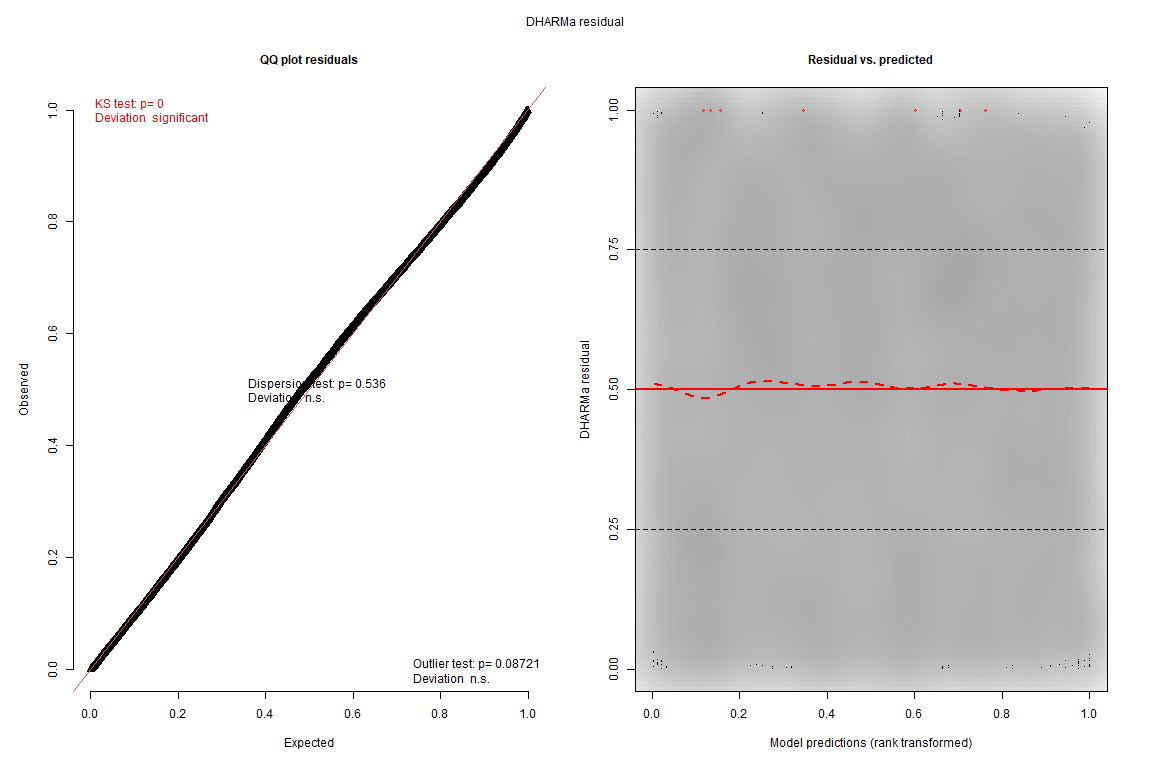


**Table S10:** Fixed effects for modelled temporal trends of macroalgae cover on individual reefs are provided as separate .csv file due to the large size. Summary parameters are on the logit scale.

**Table S11:** Hyperparameters for modelled temporal trends of macroalgae cover on individual reefs are provided as separate .csv file due to the large size. Summary parameters are on the logit scale.

**Fig S6:** DHARMa residual diagnostics for modelled temporal trends of macroalgae cover for individual reefs where modelled macroalgae abundance exceeded 14% in at least one survey year (n=12 reefs). (a) Centipede Reef, (b)  Davies Reef, ( c) Farquharson Reef, (d) Gannet Cay, ( e) Green Island, (f) Havannah Island, (g) Hyde Reef, (h) Low Isles, (i) Reef 20-104, (j)  Reef 21-064, (k) Reef 21-529, (l) Reef 21-550.

1. Centipede Reef


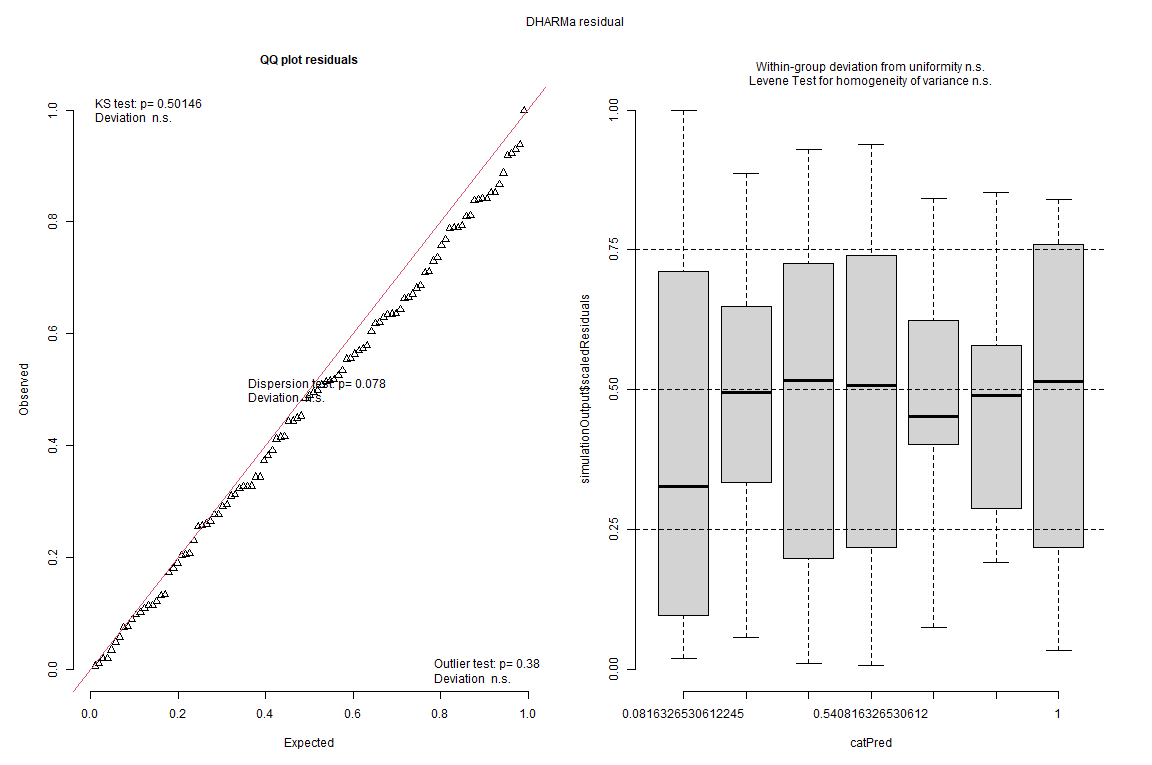


1. Davies Reef


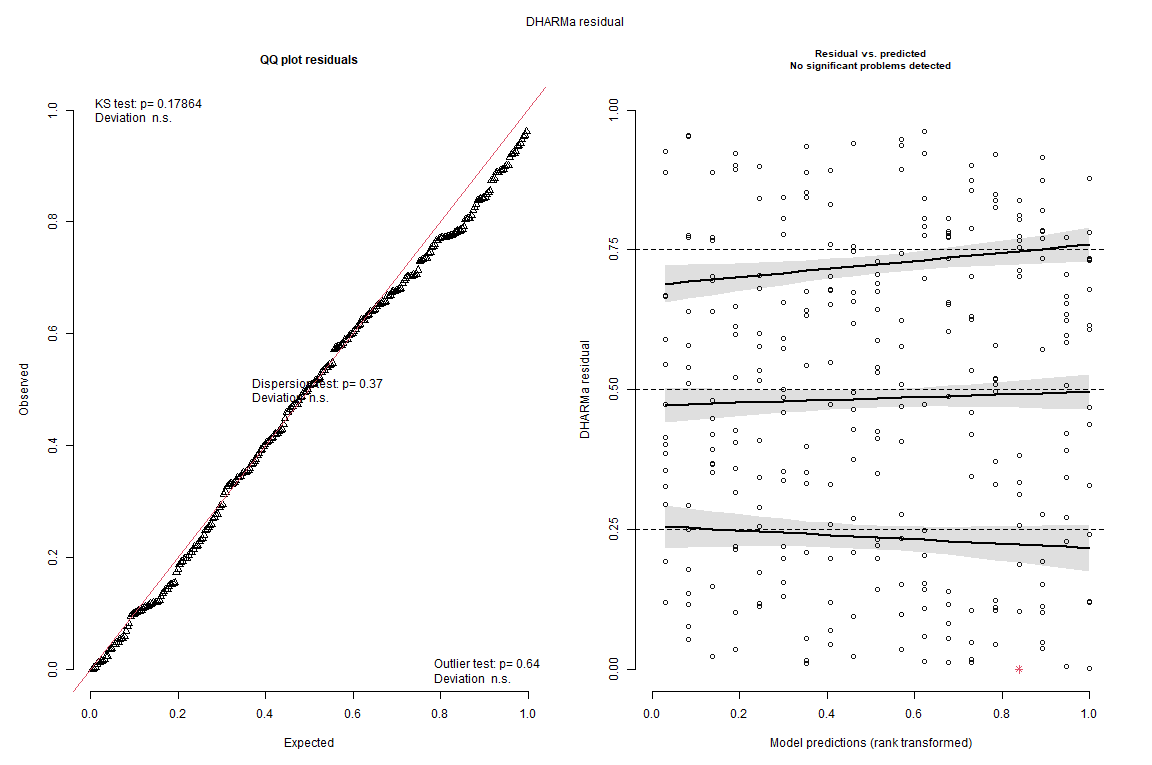


1. Farquharson Reef


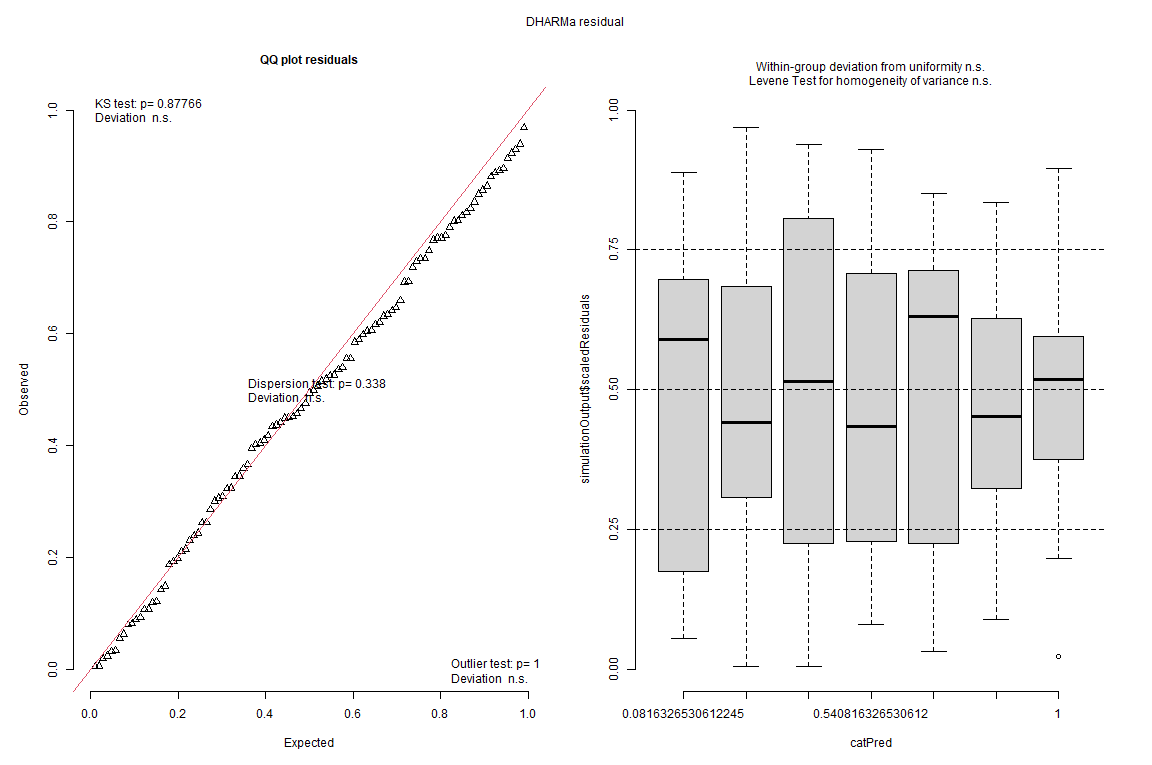


1. Gannet Cay


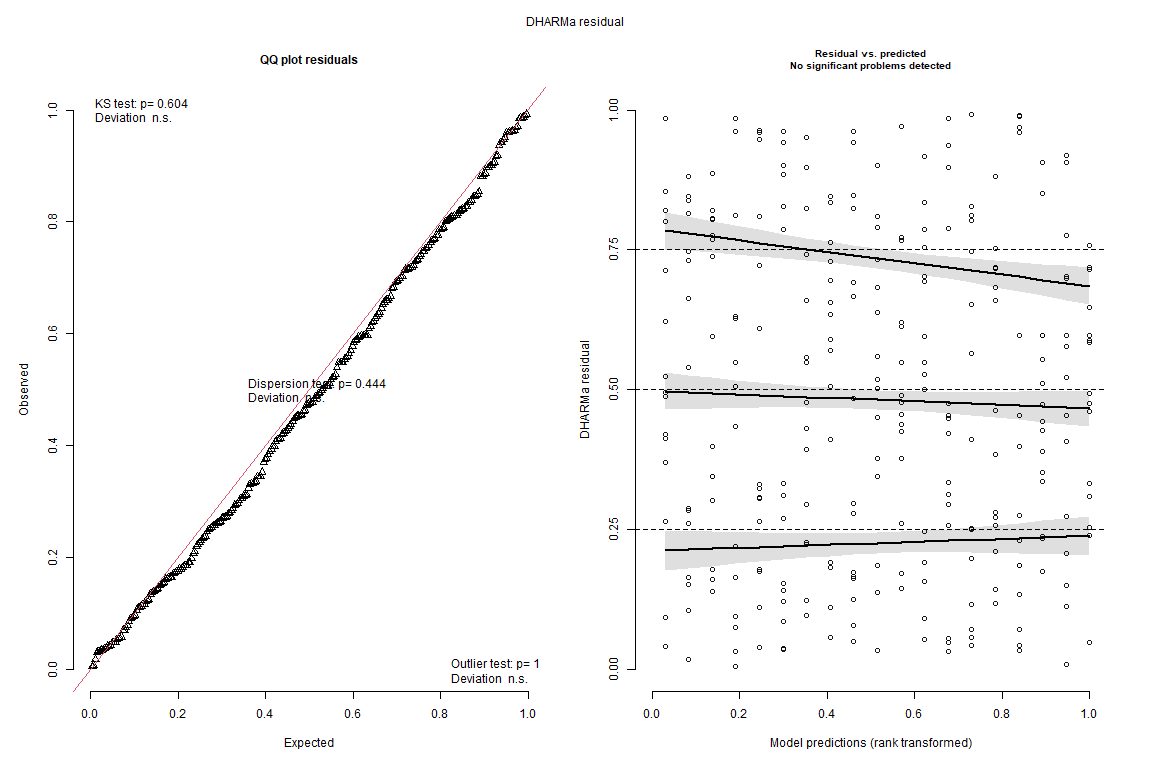


1. Green Island


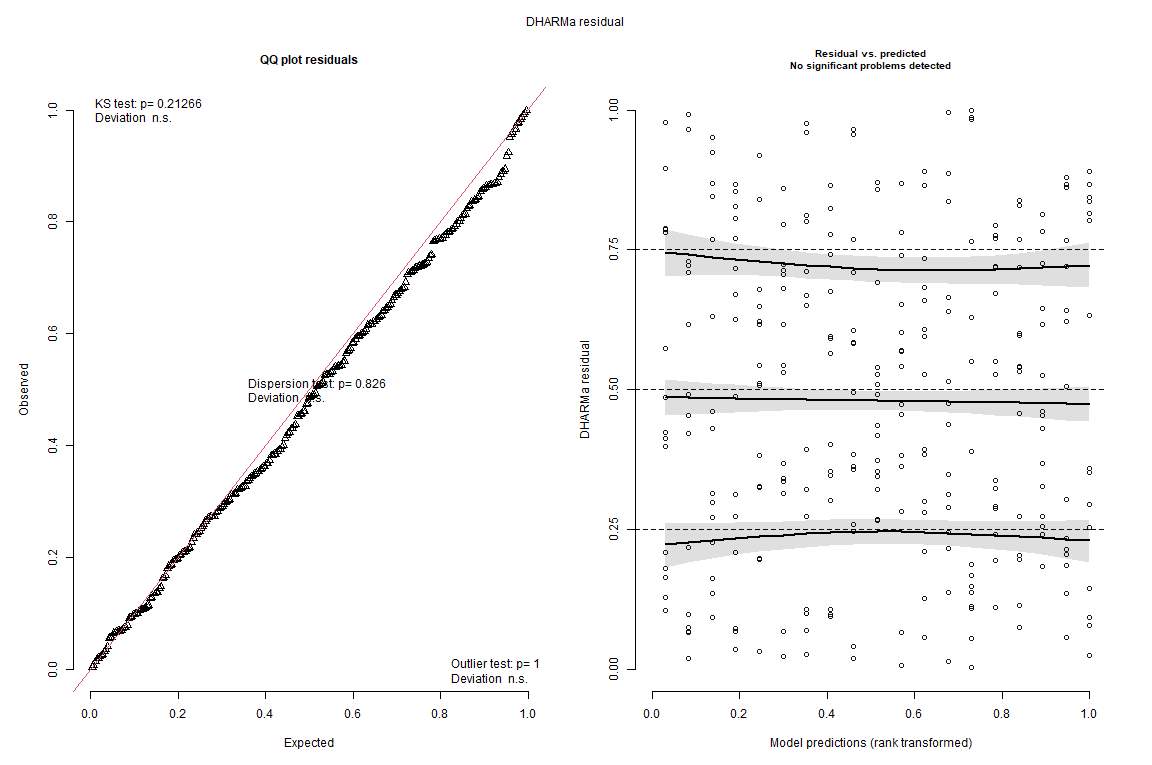


1. Havannah Island


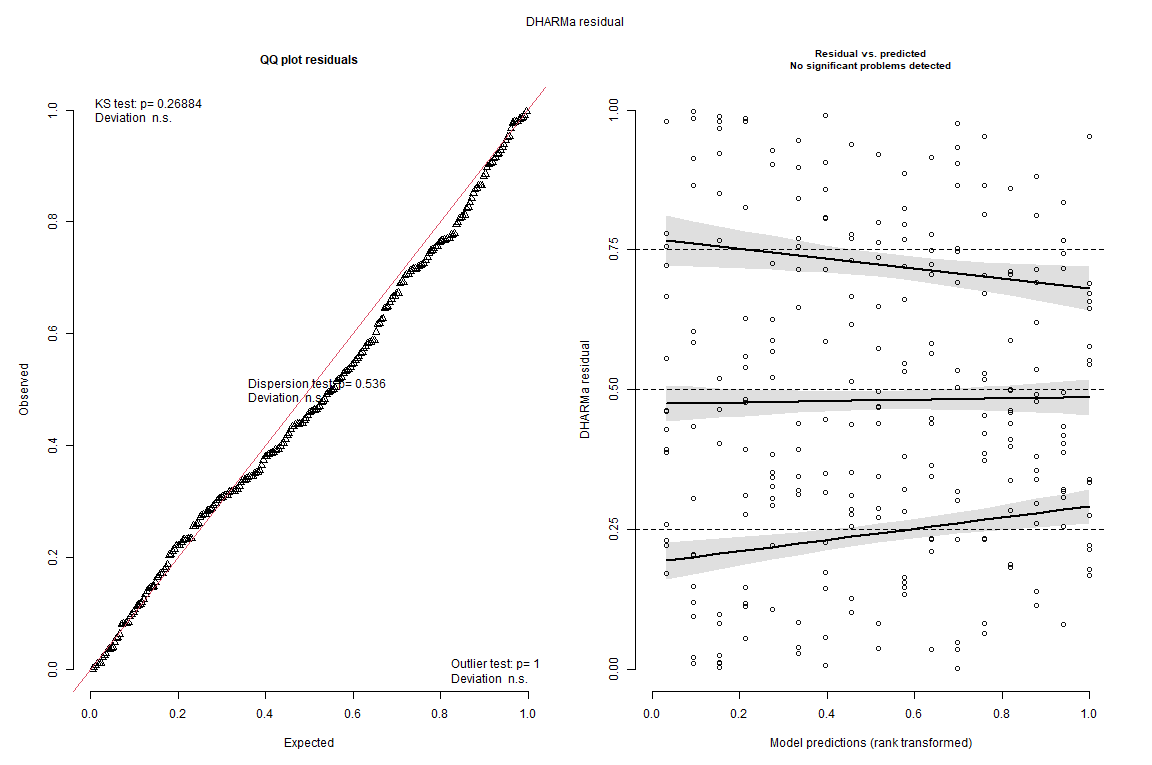


1. Hyde Reef


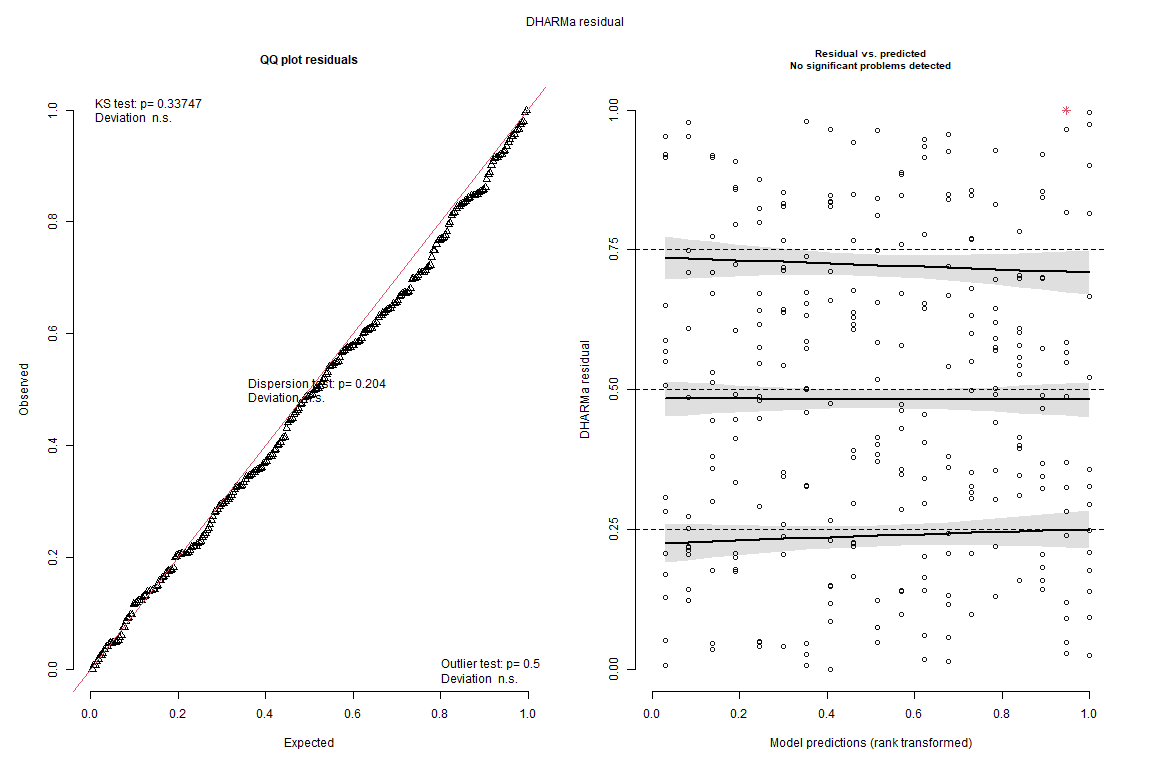


1. Low Isles


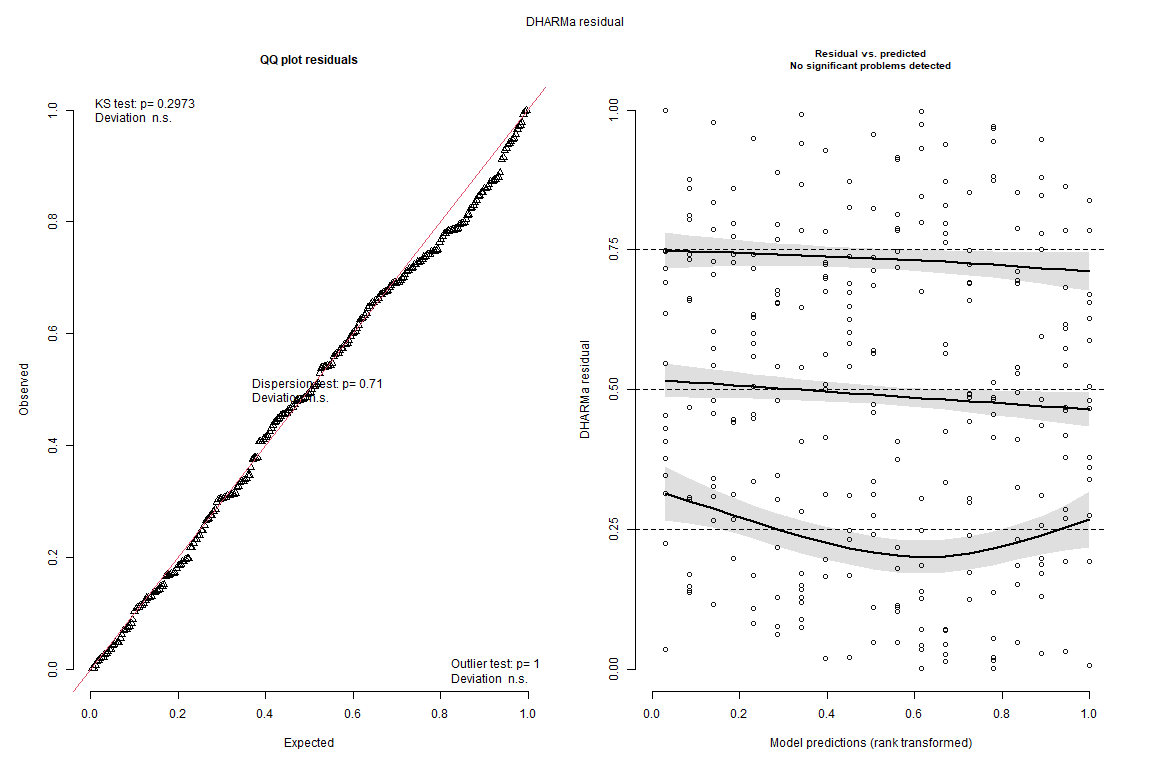


1. Reef 20-104


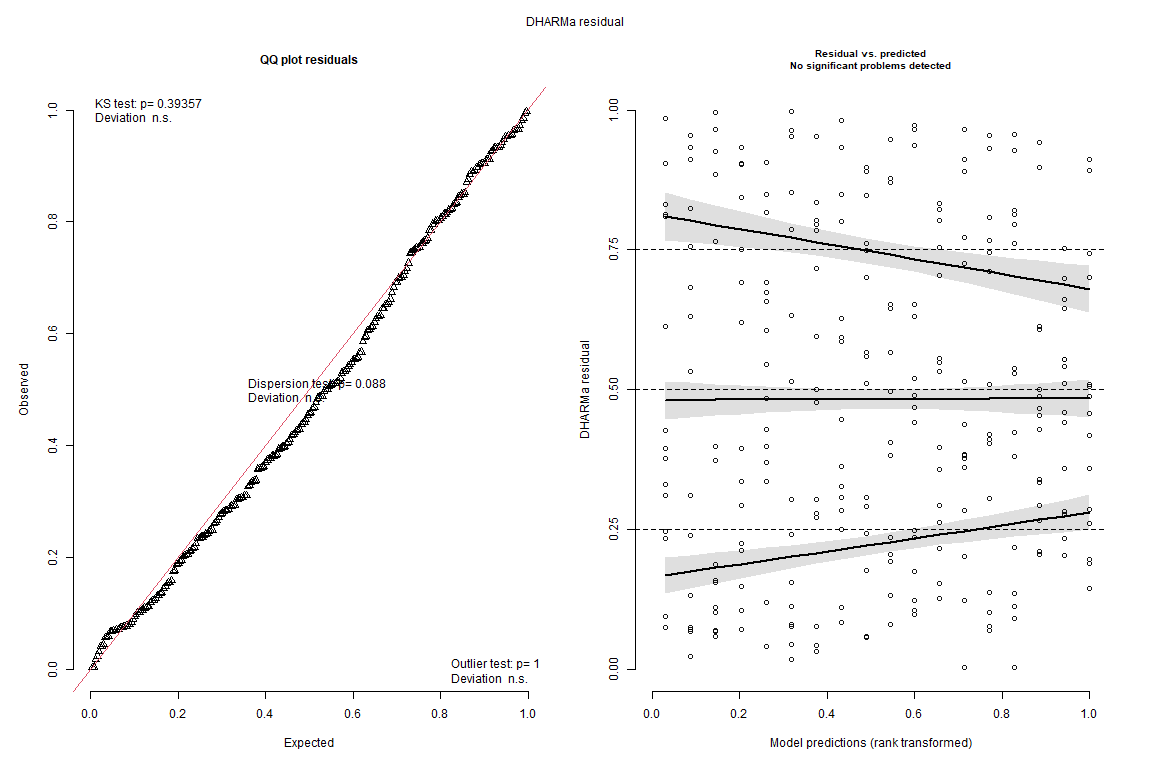


1. Reef 21-064


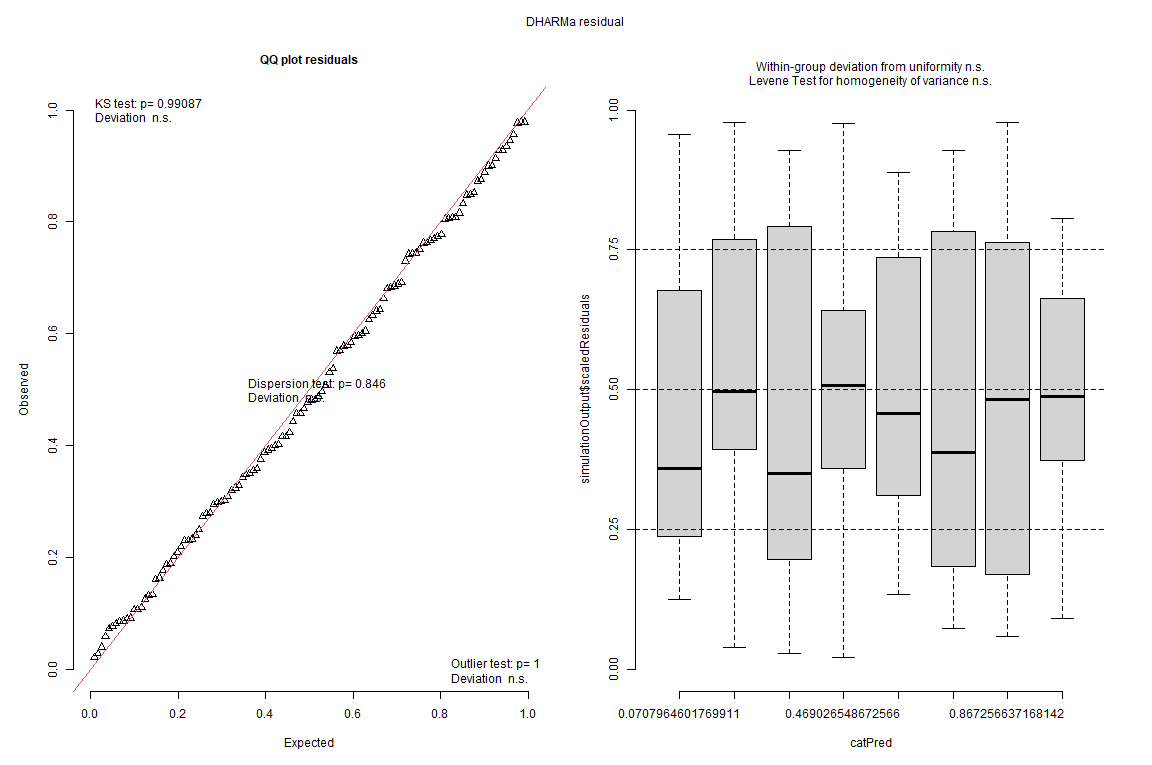


1. Reef 21-529


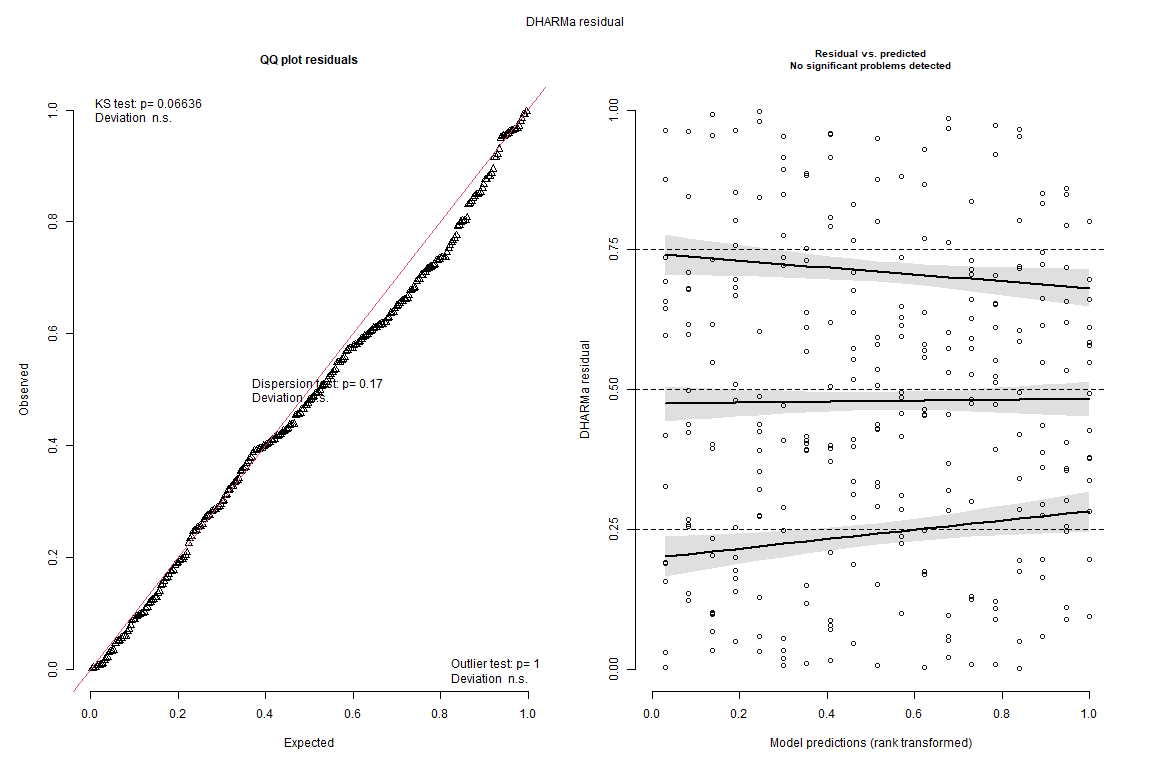


1. Reef 21-550


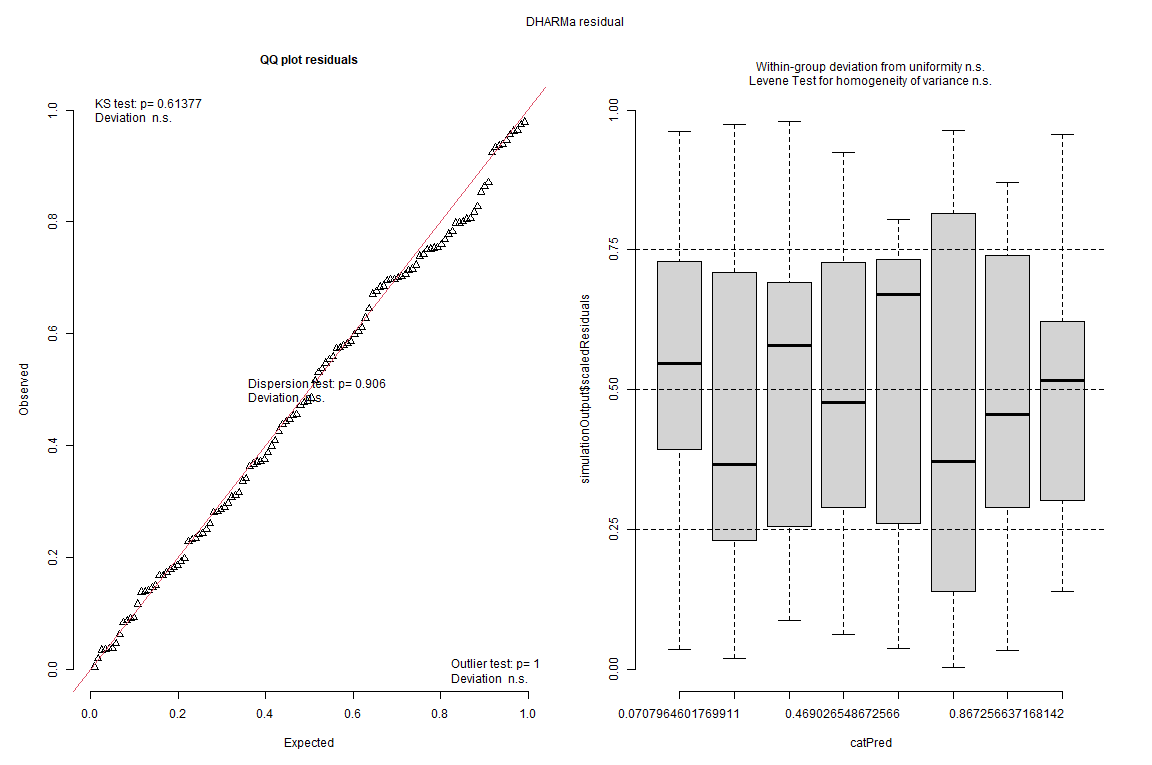

Supplement: S1 File — (DOCX) [file pone.0279699.s001.docx]
